# Anandamide Inhibits Vascular Smooth Muscle Migration, Endothelial Adhesion Protein Expression and Monocyte Adhesion of Human Coronary Artery Cells

**DOI:** 10.3390/cells13242108

**Published:** 2024-12-19

**Authors:** Elane Blessing, Elisa Teichmann, Burkhard Hinz

**Affiliations:** Institute of Pharmacology and Toxicology, Rostock University Medical Center, Schillingallee 70, 18057 Rostock, Germany; blessingelane@aol.de (E.B.);

**Keywords:** anandamide, 2-arachidonoylglycerol, endocannabinoid, human coronary artery, smooth muscle cells, endothelial cells, migration, vascular cell adhesion molecule-1, monocyte adhesion

## Abstract

Endocannabinoids have been shown to play a complex role in the pathophysiology of a number of cardiovascular disorders. In the present study, the effects of the two major endocannabinoids anandamide (AEA) and 2-arachidonoylglycerol (2-AG) were investigated in human coronary artery smooth muscle cells (HCASMC) and human coronary artery endothelial cells (HCAEC) with regard to potential atheroprotective and anti-inflammatory effects. In HCASMC, AEA showed an inhibitory effect on platelet-derived growth factor-induced migration, but not proliferation, independent of major cannabinoid-activatable receptors (CB_1_, CB_2_, TRPV1), while 2-AG left both responses unaffected. In HCAEC, AEA at concentrations of 6 and 10 µM significantly inhibited the interleukin (IL)-1β- and lipopolysaccharide (LPS)-stimulated expression of vascular cell adhesion molecule-1 (VCAM-1) and LPS-induced intercellular adhesion molecule-1 (ICAM-1), again independently of the abovementioned receptors. Corresponding effects were observed to a lesser extent in the presence of 2-AG, in most cases not significantly. The detection of activated phosphoproteins as well as experiments with inhibitors of corresponding signaling pathways suggest that AEA interferes with IL-1β-induced VCAM-1 expression via inhibition of protein kinase B/Akt and Src kinase activation and attenuates LPS-induced VCAM-1 and ICAM-1 expression via inhibition of signal transducer and activator of transcription 3 (STAT3) phosphorylation. As expected, AEA also led to a significant inhibition of monocyte adhesion to IL-1β- and LPS-stimulated HCAEC, with siRNA experiments confirming the functional role of VCAM-1 and ICAM-1 in this assay. 2-AG showed a comparatively weaker but, in the case of LPS stimulation, still significant inhibition of adhesion. In summary, the results emphasize the potential of AEA as a protective regulator of atherosclerotic and inflammation-related changes in HCASMC and HCAEC and encourage further corresponding preclinical studies with this endocannabinoid.

## 1. Introduction

The endocannabinoid system in the original definition consists of the cannabinoid receptors CB_1_ and CB_2_, its endogenous receptor ligands anandamide (*N*-arachidonoylethanolamine, AEA) and 2-arachidonoylglycerol (2-AG) as well as the enzymes that synthesize and degrade these so-called endocannabinoids. In the meantime, this system has been expanded to include further components in the sense of an endocannabidiome, such as additional cannabinoid-activatable receptors (for review, see [1,2,3]). Thereby, effects of endogenous and exogenous cannabinoids can also be triggered independently of the aforementioned receptors [4,5].

The cardiovascular effects of endocannabinoids are complex and involve modulation of the autonomic flow of the central and peripheral nervous system as well as direct effects on the myocardium and the vascular system (for review, see [6,7]). To date, rather contradictory results have been published on the impact of the endocannabinoid system on the myocardium and coronaries. On the one hand, knocking out the AEA-degrading enzyme fatty acid amidohydrolase (FAAH), resulting in an increase in AEA concentrations, led to reductions in age-related cardiac dysfunction, myocardial nitrative stress, the expression of inflammatory genes and apoptosis in mice [8]. Similarly, CB_2_ receptor activation attenuated the tumor necrosis factor (TNF)-α-induced inflammatory response in human coronary artery endothelial cells (HCAEC) and the lipopolysaccharide (LPS)-induced vascular inflammatory response in mice [9]. On the other hand, AEA was shown to cause the death of HCAEC [10] and cardiomyocytes [11] via activation of CB_1_ receptors, which was accompanied by the formation of reactive oxygen species (ROS) and activation of mitogen-activated protein kinases (MAPKs). In a number of preclinical animal models of heart failure [11], atherosclerosis [12] and diabetic retinopathy [13], it has also been demonstrated that deletion of the CB_1_ receptor or its pharmacological inhibition has a reducing effect on vascular or myocardial inflammation. Similarly, a CB_1_ receptor antagonist has been found to attenuate human coronary artery smooth muscle cell (HCASMC) proliferation and migration, two key events in the progression of atherosclerosis and development of restenosis [14]. Finally, an increase in 2-AG levels led to an impairment of endothelial repair and proliferation of HCAEC, and 2-AG also facilitated adhesion of monocytes to endothelial cells [15].

Recent studies with the non-psychoactive phytocannabinoids cannabidiol (CBD) and tetrahydrocannabivarin (THCV) have shown that protective anti-inflammatory cannabinoid effects on HCAEC can also be mediated independently of activation of CB_1_ and CB_2_ receptors, as demonstrated by the inhibitory effect on the expression of monocyte adhesion molecules [16,17]. Irrespective of the contradictory effects of CB_1_ and CB_2_ activation shown above, corresponding receptor-independent effects are also conceivable for endocannabinoids. Accordingly, the present study investigated the effect of AEA and 2-AG on the expression of vascular cell adhesion molecule-1 (VCAM-1) and intercellular adhesion molecule-1 (ICAM-1) induced by two different inflammatory stimuli, interleukin (IL)-1β and LPS, and on the adhesion of human monocytes to appropriately treated HCAEC. According to various preclinical and clinical studies, VCAM-1 and ICAM-1 promote the recruitment, adhesion and transmigration of leukocytes into inflamed tissue and ultimately promote atherosclerosis (for review, see [18,19,20]). In addition, platelet-derived growth factor (PDGF)-induced proliferation and migration of HCASMC, two crucial events in the development of atherosclerosis and restenosis (for review, see [21,22]), were investigated with regard to their modulation by the two endocannabinoids. The effects shown here demonstrate that AEA, tested at non-toxic concentrations, has protective effects on pro-atherosclerotic and pro-inflammatory changes in HCASMC and HCAEC that are independent of the AEA-activated receptors CB_1_, CB_2_ and transient receptor potential vanilloid 1 (TRPV1). These results should encourage further preclinical studies with this compound.

## 2. Materials and Methods

### 2.1. Chemicals and Reagents

AEA (#90050), 2-AG (#62160), AM251 (#71670), AM630 (#10006974), LY294002 (#70920) and Stattic (#14590) were purchased from Cayman Chemical (Ann Arbor, MI, USA). Aprotinin, bromophenol blue, calcein acetoxymethyl ester (calcein-AM; #17783), capsazepine (#C191), Hoechst 33342, hydrogen peroxide solution (H_2_O_2_, 30%), IL-1β human (#SRP3083), LPS from *E. coli* O111:B4 (#L2630), luminol, orthovanadate, paraformaldehyde (PFA), p-coumaric acid, phenylmethylsulfonyl fluoride (PMSF), poly-D-lysine hydrobromide (PDL) and PP1 (#P0040) were obtained from Sigma-Aldrich (Taufkirchen, Germany). Gibco^TM^ penicillin-streptomycin, Gibco^TM^ trypan blue solution (0.4%), Gibco^TM^ trypsin-EDTA, PDGF-BB human (#PHG0041), Lipofectamine™ RNAiMAX (#13778075) transfection reagent and Opti-MEM^TM^ I Reduced Serum Medium (#31985062) were purchased from Thermo Fisher Scientific (Schwerte, Germany). Aqua ad injectabilia was supplied by Braun Melsungen (Melsungen, Germany), and methanol was acquired from J. T. Baker (Griesheim, Germany). Dithiothreitol (DTT), leupeptin and Phalloidin-iFluor^TM^ 555 Conjugate were supplied by Biomol (Hamburg, Germany). 4-(2-hydroxyethyl)-1-piperazineethanesulfonic acid (HEPES) and 2-mercaptoethanol were bought from Ferak Berlin (Berlin, Germany). Acetic acid, dimethyl sulfoxide (DMSO), ethylenediaminetetraacetic acid (EDTA), glycerin, glycine, hydrochloric acid 37% (HCl), Ponceau S, sodium chloride (NaCl), sodium hydroxide (NaOH), sodium dodecyl sulfate (SDS) ultrapure, Tris ultrapure and Tris hydrochloride (Tris HCl) were acquired from AppliChem (Darmstadt, Germany). Acrylamide (Rotiphorese^®^ Gel, 30%), albumin (IgG-free), ammonium peroxydisulphate (APS), crystal violet, *N*,*N*,*N*’,*N*’-tetramethylethylenediamine (TEMED), Triton^®^ X-100, Tween^®^ 20 and ammonium chloride (NH_4_Cl) were purchased from Carl Roth (Karlsruhe, Germany). Dulbecco’s phosphate-buffered saline (DPBS) and fetal calf serum (FCS) were bought from PAN-Biotech (Aidenbach, Germany). Non-fat milk (NFM) powder and VECTASHIELD Antifade Mounting Medium were obtained from Bio-Rad Laboratories (Munich, Germany) and Biozol Diagnostica (Eching, Germany), respectively.

### 2.2. Cell Culture

Experiments were performed using primary human coronary artery smooth muscle cells (HCASMC, #C-12511) or primary human coronary artery endothelial cells (HCAEC, #C-12221). Both cell types were derived from the same donor and purchased from Promocell (Heidelberg, Germany) together with the respective media and supplements. Cryopreserved HCASMC were supplied at passage two and HCAEC at passage three, both stored in liquid nitrogen.

HCASMC were maintained in Smooth Muscle Cell Basal Medium 2 (#C-22262) supplemented with Growth Medium 2 Supplementpack containing 5% fetal calf serum (FCS), human recombinant epidermal growth factor (EGF; 0.5 ng/mL), human recombinant basic fibroblast growth factor (bFGF; 2 ng/mL) and human recombinant insulin (5 µg/mL). HCAEC were cultured in Endothelial Cell Basal Medium MV2 (#C-22221) complemented with Growth Medium MV2 Supplementpack containing 5% FCS, hydrocortisone (0.2 μg/mL), ascorbic acid (1 μg/mL), human recombinant vascular endothelial growth factor 165 (VEGF; 0.5 ng/mL), human recombinant insulin-like growth factor (long R3 IGF 20 ng/mL), human recombinant bFGF (10 ng/mL) and human recombinant EGF (5 ng/mL). Both types of basal medium were ordered separately with their individual Supplementpacks (#C-22162 for HCASMC, #C-22121 for HCAEC) and prepared shortly before use. In addition, 100 U/mL penicillin and 100 µg/mL streptomycin were added to both types of media. The prepared media compositions are hereafter designated as growth medium. Cells were maintained in T75 or T175 culture flasks at a density of 5000 to 10,000 cells/cm^2^. The media was changed every two to three days.

The Promocell DetachKit (#C-41220), designed for the gentle and effective detachment of adherent primary human cells, was used for subcultivation. It consists of the three components: HEPES BSS (HEPES buffered saline solution), trypsin/EDTA solution (ratio 0.04%/0.03%) and trypsin neutralization solution (TNS).

Adhesion experiments were performed using HCAEC and the human monocytic cell line THP-1 (ACC16, RRID:CVCL_0006) purchased from the German Collection of Microorganisms and Cell Cultures (DSMZ, Braunschweig, Germany). The suspension cell line THP-1 was cultured in T75 suspension cell flasks in RPMI 1640 medium (#BE12-702F, with L-Glutamine, Lonza, Verviers, Belgium) supplemented with 10% FCS, 100 U/mL penicillin and 100 µg/mL streptomycin. All primary cells and cell lines were placed in an incubator with humidified air at 37 °C containing 5% CO_2_.

All experiments, except for the scratch wound assay, were performed in serum-reduced medium without any supplements other than 2% FCS, 100 U/mL penicillin and 100 µg/mL streptomycin. Final concentrations of solvents varied by experiment, but never exceeded 0.1% (*v*/*v*) for aqua ad iniectabilia (IL-1β, LPS), 0.025% (*v*/*v*) for 0.1 M acetic acid/0.1% (*w*/*v*) albumin (PDGF-BB) and 0.2% (*v*/*v*) for DMSO (AEA, 2-AG, AM251, AM630, capsazepine, LY294002, PP1, Stattic). After seeding, the cells were allowed to adhere for 24 h and were washed with DPBS before treatment. Equal solvent concentrations were adjusted in vehicle controls of the respective experiments.

### 2.3. Cellular Viability Assays

To assess the influence of endocannabinoids on cellular metabolic activity, a colorimetric WST-1 assay was performed. To this end, 5000 cells/well were seeded in 96-well plates and maintained in growth medium for 24 h. While HCAEC were subsequently stimulated with the compounds or vehicle, HCASMC were pretreated in serum-reduced medium for 24 h prior the addition of the test substance or vehicle. After the indicated incubation time, a water-soluble tetrazolium salt 4-[3-(4-iodophenyl)-2-(4-nitrophenyl)-2H-5-tetrazolio]-1,3-benzenedisulfonate (WST-1) was added at a final dilution of 1:10. Metabolic activity was determined after 1 h of incubation by measuring the absorbance at 450 nm/690 nm with a microplate reader. WST-1 is converted to formazan by mitochondrial dehydrogenases. The metabolic reaction depends on NAD(P)H formation in viable cells. Consequently, the number of viable cells correlates with the activity of mitochondrial dehydrogenases and formazan dye production.

The cell number was determined following the absorbance measurement. For this purpose, the cells were fixed with ice-cold absolute ethanol for 24 h and then incubated in 100 µL of crystal violet staining solution (0.1% (*w*/*v*) crystal violet in 10% (*v*/*v*) ethanol) for 30 min. The surplus dye was then carefully rinsed off, the remaining dye was resolved with 10% (*v*/*v*) acetic acid, and the absorbance at 570 nm was determined using a microplate reader. The amount of crystal violet staining in the assay is directly proportional to the cell biomass and allows an assessment of the cell number.

### 2.4. BrdU Incorporation Assay

The BrdU cell proliferation ELISA from Roche Diagnostics (Mannheim, Germany) was used to evaluate the effects of the test substances on cell proliferation. This sensitive, colorimetric immunoassay is based on the incorporation of 5-bromo-2-deoxyuridine (BrdU) into the newly synthesized DNA of actively proliferating cells. BrdU acts as a thymidine analog and replaces thymidine. The immunochemical detection of incorporated BrdU enables the assessment of proliferating cells. Therefore, HCASMC were seeded in 96-well plates at a density of 5000 cells/well and allowed to adhere in growth medium for 24 h. The cells were cultured in serum-reduced medium for a further 24 h before treatment. Next, the cells were stimulated with 25 ng/mL PDGF-BB or vehicle (0.025% 0.1 M acetic acid/0.1% (*w*/*v*) BSA) and treated with increasing concentrations of the test substances or vehicle. The stimulation was repeated after 3 days. The BrdU reagent was added 24 h before analysis, which was performed after 144 h (6 days), as instructed by the manufacturer.

### 2.5. Scratch Wound Assay

To evaluate the influence of endocannabinoids on PDGF-induced migration of HCASMC, scratch wound experiments were performed. For this purpose, HCASMC were seeded in 24-well plates at 50,000 cells/well and grown to confluence in complete growth medium. The cells were cultured in serum-reduced medium (0.5% FCS, no growth factors) for a further 24 h. A 10 µL pipette tip was used as a tool to create a scratch. The cell monolayer was scraped in a straight line, and the debris was washed away with DPBS. The marker points of the scratch were created as a reference. The cells were then incubated with the test compounds in reduced medium.

Light microscopic images of the scratch area were taken 0 h, 6 h and 24 h after stimulation using a 5× objective (AxioVert.A1, Carl Zeiss, Oberkochen, Germany). Image analysis was accomplished by the program ZEN 2 blue edition (version 2.0.0.0) from Carl Zeiss. The following formula was applied to calculate the wound closure: [(wound area after 0 h − wound area after 6 h or 24 h)/wound area after 0 h] × 100%. To investigate possible involvement of cannabinoid-modulated receptors, preincubation with AM251 (CB_1_ antagonist), AM630 (CB_2_ antagonist) or capsazepine (TRPV1 antagonist) at 1 μM concentrations or vehicle was performed 1 h prior to coincubation with 10 µM AEA or vehicle in combination with 25 ng/mL PDGF-BB or vehicle for an additional 24 h.

After completion of the scratch wound experiment, the medium was aspirated, and the cells were fixed with ice-cold absolute ethanol for 24 h. Staining was performed for 30 min in 100 µL of crystal violet staining solution (0.1% (*w*/*v*) crystal violet in 10% (*v*/*v*) ethanol), and a final wash step was carried out with DPBS.

### 2.6. Quantitative Reverse Transcriptase Polymerase Chain Reaction (qRT-PCR)

Using complete growth medium, 200,000 HCAEC/well were seeded into 6-well plates and grown for 24 h. After a washing step with DPBS, the cells were exposed to the respective test substances or vehicles in reduced medium for a further 24 h. The supernatant was then separated from the cells and collected on ice in a Falcon tube. The cells were rinsed with warm DPBS, detached by trypsin/EDTA and harvested in the medium supernatant. In this way, both adherent and non-adherent cells were collected. The cell suspension was centrifuged at 200× *g*, 4 °C for 7 min, and the cell pellets were washed with DPBS and centrifuged again at 250× *g*, 4 °C for 5 min. Following cell lysis, total RNA was isolated with the RNeasy Mini Kit from Qiagen (Hilden, Germany), as instructed by the manufacturer. The NanoDrop^TM^ OneC Microvolume UV-VIS spectrophotometer (Thermo Fisher Scientific) was used to assess total RNA concentrations.

Changes in mRNA expression were analyzed using the Applied Biosystems Taq-Man^®^ Gene Expression Assay (VCAM-1: Hs01003372_m1; ICAM-1: Hs00164932_m1; both FAM-MGB) and the Applied Biosystems^®^ TaqMan^®^ RNA-to-CT™ 1-Step Kit (Thermo Fisher Scientific), as described by the manufacturer. Peptidylprolyl isomerase A (PPIA: Hs999904_m1; VIC-MGB) served as an internal standard to normalize VCAM-1 and ICAM-1 mRNA levels.

### 2.7. Total Cellular Protein Isolation

For protein expression analysis, 200,000 HCAEC/well were seeded in 6-well plates and cultured in complete growth medium for 24 h. Treatment with test substances or vehicles was carried out in serum-reduced medium containing no growth factors. After the respective incubation period, cells were detached with trypsin/EDTA and collected on ice together with the cell culture medium. After centrifugation for 7 min at 200× *g*, 4 °C, the supernatant was discarded, and the cell pellet was resuspended in 1 mL DPBS. The cell suspension was transferred in a 1.5 mL tube and centrifuged at 250× *g*, 4 °C for 5 min. Finally, the DPBS supernatant was aspirated, and the cell pellet was carefully resuspended in lysis buffer (50 mM HEPES, 150 mM NaCl, 1 mM EDTA, 1% (*v*/*v*) Triton^®^ X-100, 10% (*v*/*v*) glycerol, 10 µg/mL aprotinin, 1 µg/mL leupeptin, 1 mM orthovanadate, 1 mM PMSF) and incubated at −20 °C for at least 30 min. After a final centrifugation step at 20,817× *g*, 4 °C for 5 min, the entire cellular protein remained in the supernatant. Finally, the supernatant was transferred to a new 1.5 mL tube and stored at −20 °C until further analysis.

To prevent early dephosphorylation, cells were scraped with a cell scraper in lysis buffer (2% (*w*/*v*) SDS, 40% (*v*/*v*) aqua ad iniectabilia, 10% (*v*/*v*) glycerol, 50% (*v*/*v*) 125 mM Tris-HCl (pH 6.8)) on ice for analysis of phosphorylated proteins and the corresponding unphosphorylated variants. The lysate was boiled at 95 °C for 10 min and then centrifuged at 20,817× *g* and 4 °C for 5 min. The supernatant, which contained all the cellular protein, was stored at −20 °C with β-mercaptoethanol. For both methods described, total protein concentration was determined using the Pierce™ Bicinchoninic Acid (BCA) Protein Assay Kit (Thermo Fisher Scientific), following the manufacturer’s protocol.

### 2.8. Western Blot Analysis

Equal amounts of protein were separated on 8%, 10% or 15% SDS polyacrylamide gels, depending on protein size. After transfer to nitrocellulose, the membranes were blocked in 5% (*w*/*v*) non-fat dry milk (NFM) in Tris-buffered saline with 0.1% (*v*/*v*) Tween^®^ 20 (TBS-T buffer). The membranes were rinsed with TBS-T buffer and incubated overnight at 4 °C with primary antibodies. VCAM-1 (#sc-13160, Santa Cruz Biotechnology, Heidelberg Germany, RRID:AB_626846), ICAM-1 (#sc-8439, Santa Cruz Biotechnology, Heidelberg Germany, RRID:AB_627123), E-selectin (#sc-137054, Santa Cruz Biotechnology, Heidelberg Germany, RRID:AB_2186681) and β-actin (#A5441, Sigma-Aldrich, RRID:AB_476744) antibodies were diluted in 1% (*w*/*v*) NFM in TBS-T buffer. Phospho-Stat3 (Tyr705) (sc-8059, Santa Cruz Biotechnology, RRID:AB_628292), Stat3 (sc-8019, Santa Cruz Biotechnology, RRID:AB_628293), phospho-IκBα (Ser32/36) (#9246, Cell Signaling Technology, RRID:AB_2267145), IκBα (#4814, Cell Signaling Technology, RRID:AB_390781), phospho-Src (Tyr416) (#6943, Cell Signaling Technology, RRID:AB_10013641) and Src (#2109, Cell Signaling Technology, RRID:AB_2106059) antibodies were diluted in 5% (*w*/*v*) NFM in TBS-T buffer. Phospho-Akt (Ser473) (#9271, Cell Signaling Technology, RRID:AB_329825), Akt (#9272, Cell Signaling Technology, RRID:AB_329827), phospho-IKKα/β (Ser176/180) (#2697, Cell Signaling Technology, RRID:AB_2079382), IKKβ (#8943, Cell Signaling Technology, RRID:AB_11024092), phospho-p38 MAPK (Thr180/Tyr182) (#9211, Cell Signaling Technology, RRID:AB_331641) and p38 MAPK (#9212, Cell Signaling Technology, RRID:AB_330713) primary antibodies were diluted in 5% (*w*/*v*) bovine serum albumin (BSA) in TBS-T buffer. For protein detection, membranes were probed with horseradish peroxidase-conjugated anti-rabbit secondary antibody (#7074, Cell Signaling Technology, RRID:AB_2099233) or anti-mouse secondary antibody (#7076, Cell Signaling Technology, RRID:AB_330924) in 1% (*w*/*v*) NFM in TBS-T buffer for 1 h at room temperature. To obtain a signal indicating antibody binding, a chemiluminescent solution (100 mM Tris-HCl (pH 8.5), 1.25 mM luminol, 200 µM p-coumaric acid, 0.09% (*v*/*v*) hydrogen peroxide) was added, resulting in an enhanced chemiluminescence reaction (ECL). The resulting signal was captured using the ChemiDoc XRS imaging system (Universal Hood II, Bio-Rad Laboratories, Hercules CA USA). Quantification was performed by densitometric analysis of band intensities using Quantity One 1-D analysis software (version 4.6.8). After visualization of antibody binding, membranes were washed with TBS-T, stripped (200 mM glycine, 500 mM sodium chloride, pH 2.5) and reprobed.

Target protein signals were normalized to the loading control signal (β-actin) and compared to the appropriate vehicle controls. Precision Plus Protein™ Dual Color Standard from Bio-Rad Laboratories served as a reference to determine the molecular weight of the bands.

### 2.9. Procedure of siRNA Transfection

Transfection of VCAM-1 siRNA, ICAM-1 siRNA or negative control siRNA was performed using Lipofectamine™ RNAiMAX transfection reagent (#13778075, Thermo Fisher Scientific) and Opti-MEM^TM^ I Reduced Serum Medium (#31985062, Thermo Fisher Scientific) in a reverse procedure, according to the manufacturer’s instructions. VCAM-1 siRNA (#GeneGlobe ID: SI03120299, NM_001078), ICAM-1 siRNA (#GeneGlobe ID: SI00004347, NM_000201) and negative control siRNA (#1022076) were purchased from Qiagen. First, Opti-MEM^TM^ I Reduced Serum Medium, Lipofectamine™ RNAiMAX and the desired siRNA were mixed in a solution and incubated for 20 min at room temperature to form a complex. The Lipofectamine™ RNAiMAX-siRNA complexes were then transferred into 6-well plates. In each well, 200,000 HCAEC were seeded in growth medium with a final siRNA concentration of 10 nM. The cells were allowed to adhere for 24 h. Afterwards, the transfection medium was removed, and the cells were washed with DPBS. Stimulation with 10 ng/mL IL-1β or 1 µg/mL LPS or vehicle was performed for 24 h. Finally, HCAEC were harvested by trypsinization for further analysis.

### 2.10. Fluorescence Microscopy-Based Monocyte Adhesion Assay

First, 8-chamber glass slides with polystyrene vessel (#354118, Falcon, Corning, NY, USA) were coated with poly-D-lysine (PDL), then HCAEC were seeded at a density of 50,000 cells/chamber and allowed to attach for 24 h in complete growth medium. Cells were gently rinsed with DPBS and exposed to compounds or vehicle for 24 h in serum-reduced medium. During the last 40 min of treatment, THP-1 monocytes were stained with 5 µM calcein-AM fluorescent dye for 30 min in an incubator and carefully washed with DPBS. Shortly before coincubation, HCAEC were washed with DPBS. Then, 100,000 labeled THP-1 cells/chamber were added to the endothelial cells for 30 min in an equal amount of serum-reduced endothelial cell medium and serum-reduced RPMI-1640 medium. Unbound monocytes were carefully removed with DPBS. Cells were then fixed with 4% (*w*/*v*) PFA for 10 min and subsequently treated with 50 mM NH_4_Cl for 5 min. After washing with DPBS, cells were permeabilized with DPBS containing 0.3% (*v*/*v*) Triton^®^ X-100 for 15 min and washed again with DPBS. To reduce the background signal, the free binding sites were blocked with 0.5% (*w*/*v*) albumin and 0.1% (*v*/*v*) Tween^®^ 20 dissolved in DPBS for 5 min.

Prior to staining, the cells were again rinsed with DPBS. This was followed by incubation with bisbenzimide (Hoechst 33342) and Phalloidin-iFluor™ 555 Conjugate, fluorescent dyes that stain nuclei and F-actin, respectively. The 1 h of fluorescent labeling was followed by a wash step. Finally, coverslips were mounted on the glass slides with VECTASHIELT Antifade Mounting Medium to prevent rapid bleaching. Fluorescence images were acquired using AxioScope.A1 (Carl Zeiss, Oberkochen, Germany) and ZEN 2 blue edition software (version 2.0.0.0). For quantification, the number of calcein-AM-positive cells was set in relation to bisbenzimide-positive cells.

### 2.11. Statistics

All values are given as means ± standard error of the mean (SEM). Calculations were obtained using GraphPad Prism 9.3.0 or an updated version (GraphPad Software, San Diego, CA, USA). Comparisons between two groups were assessed with the unpaired two-tailed Student’s t test. Comparisons between multiple groups and a vehicle group were performed using one-way ANOVA with a Dunnett post hoc test. When multiple groups were compared with more than one group of interest, a one-way ANOVA with a Bonferroni post hoc test was performed. In the case of a Bonferroni post hoc test, the determination of statistical significance was limited to the groups of interest for reasons of clarity of presentation. Repeated measures (RM) one-way ANOVA was performed to compare the means of matched groups. *p* values of ≤0.05 were considered significant.

## 3. Results

### 3.1. AEA and 2-AG Are Well Tolerated at the Concentrations Used in HCASMC

With our first approach, we aimed to ensure that the concentrations of AEA and 2-AG used had no effect on the viability of HCASMC. Therefore, a colorimetric WST-1 assay followed by crystal violet staining was performed. The combination of both assays provides reliable results for the evaluation of cytotoxicity. The experimental procedure for each endocannabinoid was conducted both under basal conditions and in combination with PDGF for 144 h (Supplementary Figure S1A–D). Growth factor treatment resulted in increases in HCASMC metabolic activity and cell number compared to the untreated control. There were no significant changes in metabolic activity or cell number of AEA- or 2-AG-treated HCASMC compared to the vehicle control (Supplementary Figure S1A–D). These results thus confirm that both endocannabinoids do not affect the viability of HCASMC at concentrations of up to 10 µM.

### 3.2. AEA and 2-AG Do Not Significantly Inhibit Basal and PDGF-Induced Proliferation of HCASMC

**Figure 1:**
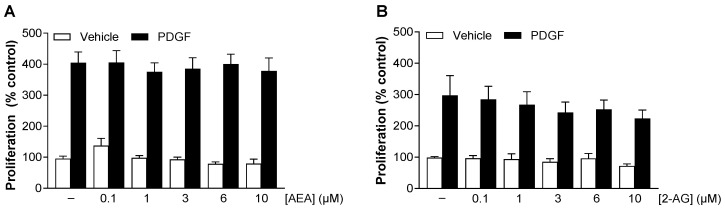
Influence of AEA (**A**) and 2-AG (**B**) on basal and PDGF-induced HCASMC proliferation. Cells were maintained in serum-reduced medium for 24 h before incubation with 25 ng/mL PDGF or its vehicle and increasing concentrations of AEA or 2-AG or its vehicle. Proliferation was assessed using the colorimetric BrdU incorporation assay after 144 h. Data are expressed as means ± SEM of *n* = 12 (4 independent experiments). Significant effects of AEA and 2-AG on basal and PDGF-induced proliferation were excluded by one-way ANOVA plus Dunnett post hoc tests.

As described above, a strong proliferation of vascular smooth muscle cells (VSMC) contributes significantly to inflammatory vascular diseases and restenosis after percutaneous interventions (for review, see [21,22]). Within this process, the vascular response to injury is characterized by a phenotypic change of medial smooth muscle cells to a synthetic/proliferative phenotype (for review, see [23]). To investigate the impact of the two endocannabinoids on PDGF-induced VSMC proliferation, a BrdU incorporation assay was performed. Cells were incubated with the indicated concentrations of AEA and 2-AG in combination with PDGF or its vehicle for 144 h. Figure 1 shows a strong induction of HCASMC proliferation in response to PDGF stimulation compared to baseline conditions. No significant changes in proliferation were observed with AEA in basal condition or in combination with PDGF at any concentration compared to the respective control (Figure 1A). Also, 2-AG did not significantly inhibit basal and PDGF-induced proliferation of HCASMC (Figure 1B), but caused a slight decrease in the PDGF effect in a concentration-dependent manner.

### 3.3. AEA Inhibits PDGF-Induced Migration of HCASMC Independent of Cannabinoid Receptors CB_1_ and CB_2_ and TRPV1 Signaling

**Figure 2:**
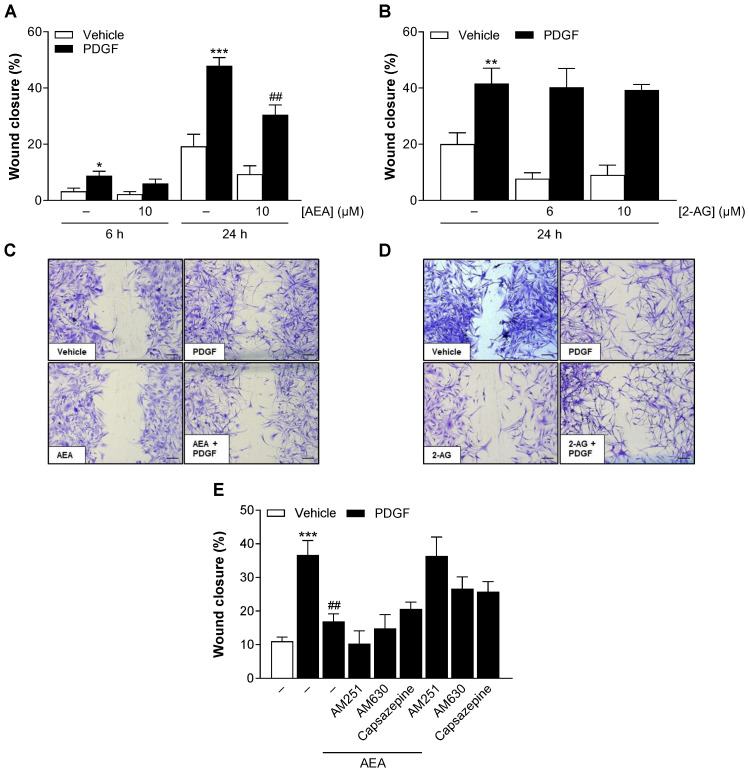
Influence of AEA (**A**,**C**,**E**) and 2-AG (**B**,**D**) on basal and PDGF-induced HCASMC migration. Cells were maintained in serum-reduced medium for 24 h before the cell monolayer was scratched in a straight line and then stimulated with 25 ng/mL PDGF or its vehicle and treated with AEA (10 µM), 2-AG (6 µM, 10 µM) or its vehicle. For the analysis of receptor signaling (**E**), cells were preincubated with the indicated receptor antagonists at 1 µM for 1 h before addition of PDGF and AEA. Light microscopic images of the scratch area were taken with a 5× objective at 0 h, 6 h (only AEA) and 24 h (**C**,**D**) after stimulation and after crystal violet staining. The scale bars indicate 200 µm. Data are expressed as means ± SEM of *n* = 8–11 (4–5 independent experiments, (**A**)), *n* = 6 (3 independent experiments, (**B**)) or *n* = 8 (4 independent experiments, (**E**)). * *p* ≤ 0.05, ** *p* ≤ 0.01, *** *p* ≤ 0.001 vs. PDGF-free vehicle control; ## *p* ≤ 0.01 vs. PDGF control; one-way ANOVA plus Bonferroni post hoc test.

In addition to proliferation, HCASMC migration also contributes significantly to the progression of chronic inflammatory vascular diseases and restenosis [24]. Therefore, the effect of AEA or 2-AG on PDGF-induced HCASMC migration was investigated using the scratch wound assay. Treatment with PDGF and the respective endocannabinoid was carried out for 24 h. An additional 6 h value was determined for AEA. Similar to the analysis of proliferation, PDGF treatment led to a significant induction of HCASMC migration compared to baseline conditions. At a concentration of 10 µM, AEA significantly suppressed PDGF-induced migration by 36% after 24 h (Figure 2A), while 2-AG showed no effect (Figure 2B). The effect of AEA was confirmed by light micrographs of the scratch area after crystal violet staining (Figure 2C). The AEA-mediated attenuation of migration occurred after 6 h but was not statistically significant (Figure 2A).

AEA is known to act as an agonist at CB_1_ and CB_2_ receptors [25] and at TRPV1 [26]. Accordingly, a possible involvement of these receptors in the AEA-mediated antimigratory effect was subsequently investigated. For this purpose, HCASMC were incubated with antagonists targeting CB_1_ (AM251), CB_2_ (AM630) or TRPV1 (capsazepine) 1 h before stimulation with AEA and PDGF for a further 24 h. Thereby, antagonist concentrations were used that have been shown to be effective in antagonizing cannabinoid effects in various cell cultures [27,28,29,30,31,32,33]. Treatment with PDGF alone resulted in a strong increase in HCASMC migration, which was significantly reduced by AEA (Figure 2E). However, the investigated receptor antagonists did not significantly reverse the inhibitory effect of AEA on PDGF-induced migration of HCASMC, making receptor involvement unlikely. In addition, the influence of the receptor antagonists themselves on PDGF-stimulated migration was investigated (Figure 2E). Thereby, attenuations of PDGF-induced migration by AM630 and capsazepine were observed, which, however, were not statistically significant. When testing the effects of the receptor antagonists used on the viability of HCASMC in the WST-1 test and crystal violet staining, statistically significant effects were excluded (Supplementary Table S1).

### 3.4. AEA and 2-AG Do Not Impair the Metabolic Activity of HCAEC at the Concentrations Used

Before studying the effect of AEA and 2-AG on endothelial pro-inflammatory responses, the impact of both endocannabinoids on the viability of HCAEC was determined. Studies with rapamycin, used in drug-eluting stents to unblock narrowed and occluded coronary arteries, have shown that unintended drug-induced inhibition of endothelial viability can lead to endothelial dysfunction and late thrombosis [34]. Therefore, a thorough analysis of novel drugs targeting the coronaries appears essential.

Cell viability was again determined using a colorimetric WST-1 assay, followed by crystal violet staining. Incubation with AEA at concentrations of 3 µM and above was found to significantly increase the metabolic activity of HCAEC by up to 18% compared to cells treated with the vehicle (Supplementary Figure S2A). However, no changes in cell number were observed in response to AEA treatment (Supplementary Figure S2B). Treatment with 2-AG up to 10 µM showed no effects on the metabolic activity of HCAEC (Supplementary Figure S2C). Noteworthy, a significant decrease in cell number was observed at 10 µM 2-AG compared to cells treated with the vehicle (Supplementary Figure S2D). However, since 2-AG showed no changes in metabolic activity at 10 µM and the cells were morphologically unremarkable, we retained this concentration in subsequent experiments.

### 3.5. At the Protein Expression Level, AEA Inhibits Basal, IL-1β- and LPS-Induced VCAM-1 as Well as Basal and LPS-Induced ICAM-1 in a Concentration-Dependent Manner

As described above, adhesion molecules induced by pro-inflammatory stimuli are essential for the recruitment of leukocytes and their adhesion to endothelial cells and thus for the development of vascular diseases (for review, see [18,19,20]). To deduce possible atheroprotective and anti-inflammatory properties of AEA or 2-AG on HCAEC, experiments were performed under basal conditions and in the presence of IL-1β or LPS. Changes in the protein expression of VCAM-1 and ICAM-1 were analyzed by Western blot technique after 24 h of cell incubation.

**Figure 3:**
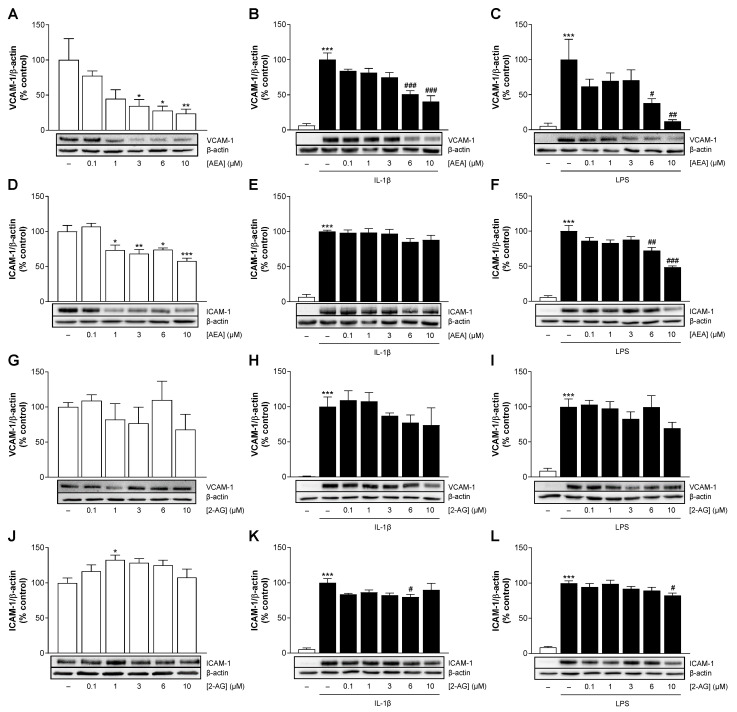
Effect of AEA (**A**–**F**) or 2-AG (**G**–**L**) on basal or IL-1β- and LPS-mediated induction of VCAM-1 and ICAM-1 protein levels in HCAEC. Protein expression values were determined by Western blot analysis and normalized to β-actin. HCAEC were incubated in the presence or absence of 10 ng/mL IL-1β and the indicated concentrations of AEA or 2-AG, as well as the respective vehicle control, for 24 h. For LPS stimulation, cells were preincubated with increasing concentrations of AEA or 2-AG or its vehicle for 1 h and then coincubated with 1 µg/mL LPS or vehicle for 24 h. The representative β-actin blots in (**B**,**E**) are identical, as the analysis of the target protein was performed on the same gel. Cells treated with vehicle (**A**,**D**,**G**,**J**) or IL-1β (**B**,**E**,**H**,**K**) or LPS (**C**,**F**,**I**,**L**) were set as 100% and served as control. Data are presented as means ± SEM of *n* = 3 (**G**), *n* = 4 (**A**,**C**), *n* = 5 (**E**,**F**,**H**), *n* = 6 (**B**,**D**,**I**,**K**), *n* = 8 (**L**) or *n* = 9 (**J**) independent experiments. * *p* ≤ 0.05, ** *p* ≤ 0.01, *** *p* ≤ 0.001 vs. vehicle control; # *p* ≤ 0.05, ## *p* ≤ 0.01, ### *p* ≤ 0.001 vs. stimulated control; one-way ANOVA plus Dunnett (**A**,**D**,**G**,**J**) or Bonferroni post hoc test (**B**,**C**,**E**,**F**,**H**,**I**,**K**,**L**).

Under basal conditions, treatment with AEA resulted in a significant concentration-dependent suppression of the protein expression of both adhesion molecules (Figure 3A,D) compared to the vehicle-treated cells. The decrease in VCAM-1 protein expression reached 76% at 10 µM AEA and was more pronounced than the suppression of ICAM-1, which was 42%. Exposure to pro-inflammatory stimuli increased the protein expression of both adhesion molecules by approximately 15- to 19-fold over basal conditions (Figure 3B,C,E,F). Simultaneous incubation of IL-1β and AEA resulted in a concentration-dependent downregulation of the induced VCAM-1 expression by up to 60%, but had no effect on ICAM-1 (Figure 3B,E). As shown in Figure 3C,F, the upregulation of VCAM-1 and ICAM-1 protein expression caused by LPS was attenuated by AEA, which intensified with increasing concentration and resulted in an 88% decrease in induced VCAM-1 expression and a 52% decrease in ICAM-1 expression at a concentration of 10 µM.

Examination of HCAEC after 2-AG treatment under basal conditions revealed no significant decrease in VCAM-1 protein levels and a slight increase in ICAM-1 protein levels (Figure 3G,J). 2-AG did not substantially alter IL-1β-induced VCAM-1 and ICAM-1 protein expression (Figure 3H,K), although there was a tendency for concentration-dependent inhibition in the case of VCAM-1. The LPS-mediated induction of VCAM-1 and ICAM-1 was attenuated by treatment with 10 µM of 2-AG by 31% and 18%, respectively (Figure 3I,L). Overall, the effects of 2-AG under pro-inflammatory conditions appeared to be much weaker than those of AEA.

Regarding the experimental protein detection, it is important to note that the exposure time of the nitrocellulose membrane to the chemiluminescent solution was longer for the analysis of unstimulated samples (Figure 3A,D,G,J) than for the stimulated samples (Figure 3B,C,E,F,H,I,K,L). This avoided signal overexposure during IL-1β- or LPS-induced VCAM-1 and ICAM-1 detection. In addition, this explains the comparatively weak signal in the vehicle controls on the membranes containing the stimulated samples (compare the first lanes of the blots in Figure 3A,D,G,J with those in Figure 3B,C,E,F,H,I,K,L).

### 3.6. Inhibition of Adhesion Molecules by AEA Under IL-1β- and LPS-Induced Conditions Is Supported by mRNA Analyses

**Figure 4:**
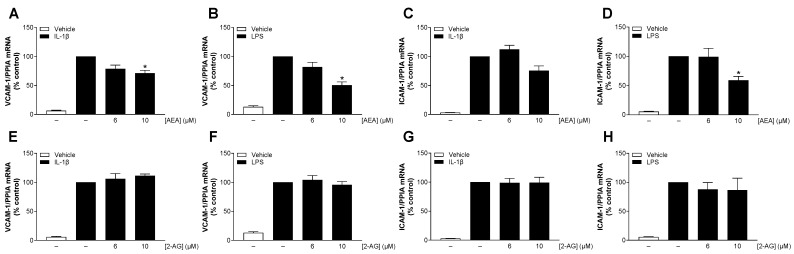
Influence of AEA (**A**–**D**) or 2-AG (**E**–**H**) on IL-1β- or LPS-induced mRNA expression of VCAM-1 or ICAM-1 in HCAEC. Cells were stimulated with 10 ng/mL IL-1β or vehicle and with the indicated concentrations of AEA or 2-AG or its vehicle for 24 h. Samples treated with LPS were preincubated with the indicated concentrations of AEA or 2-AG for 1 h before being coincubated with 1 µg/mL LPS or vehicle for a further 24 h. Expression of the indicated genes were analyzed by qRT-PCR. Peptidylprolyl isomerase A (PPIA) was used as an internal standard (housekeeping gene). Cells treated with IL-1β or LPS were used as control (set as 100%). Data are shown as means ± SEM of *n* = 3 independent experiments. * *p* ≤ 0.05 vs. IL-1β or LPS control; RM one-way ANOVA plus Dunnett post hoc test.

Next, we performed qRT-PCR to investigate possible effects of AEA or 2-AG on the mRNA levels of VCAM-1 and ICAM-1 in stimulated HCAEC. Similar to protein analysis, incubation with IL-1β or LPS was performed for 24 h to achieve an inflammatory state in the cells. Exposure to IL-1β or LPS resulted in a strong increase in VCAM-1 and ICAM-1 mRNA levels by 8–17-fold and 18–33-fold, respectively, compared to baseline conditions (Figure 4). The increase in VCAM-1 and ICAM-1 mRNA expression was inhibited by AEA treatment at a concentration of 10 µM (Figure 4A–D), with the statistical significance of these events coinciding with the protein data. Likewise, AEA-mediated VCAM-1 suppression was more pronounced with LPS than with IL-1β stimulation, consistent with the protein data. In contrast to AEA, treatment with 2-AG had no inhibitory effect at all on the induced VCAM-1 and ICAM-1 mRNA levels (Figure 4E–H).

### 3.7. Effect of AEA and 2-AG on the Protein Expression of E-Selectin

**Figure 5:**
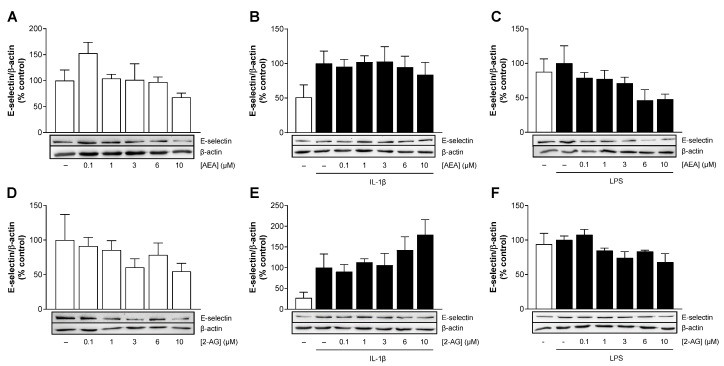
Effect of AEA (**A**–**C**) or 2-AG (**D**–**F**) on basal, IL-1β- or LPS-mediated protein expression of E-selectin in HCAEC. Cells were incubated with 10 ng/mL IL-1β or its vehicle and the indicated concentrations of AEA or 2-AG or its vehicle for 24 h. For analysis under LPS conditions, cells were preincubated with increasing concentrations of AEA or 2-AG or its vehicle for 1 h and then coincubated with 1 µg/mL LPS or vehicle for 24 h. Cells treated with vehicle (**A**,**D**) or IL-1β (**B**,**E**) or LPS (**C**,**F**) alone were used as control and set as 100%. Data are presented as means ± SEM of *n* = 3 (**A**,**C**,**E**,**F**) or *n* = 4 (**B**,**D**) independent experiments. Statistical significance for endocannabinoid or IL-1β/LPS effects were excluded using one-way ANOVA plus Dunnett (**A**,**D**) or Bonferroni post hoc test (**B**,**C**,**E**,**F**).

In addition to VCAM-1 and ICAM-1, which are the focus of the present study, we also addressed the regulation of another adhesion molecule, E-selectin, for comparative purposes. As shown in Figure 5B,E, treatment with IL-1β resulted in a 2–3.7-fold increase in E-selectin protein expression compared to cells treated with the vehicle. In contrast, LPS-stimulated HCAEC exhibited no changes in E-selectin protein expression compared to vehicle-treated controls (Figure 5C,F). Both endocannabinoids tended to attenuate E-selectin protein expression at baseline and in the presence of LPS (Figure 5A,C,D,F). While AEA showed no changes in IL-1β-induced E-selectin expression (Figure 5B), 2-AG appeared to enhance IL-1β-induced E-selectin protein expression (Figure 5E). Since none of the endocannabinoids had a profound effect on the induced protein expression of E-selectin, we excluded this adhesion molecule from further analysis.

### 3.8. AEA Reduces VCAM-1 and ICAM-1 Protein Levels in HCAEC Independently of the Cannabinoid Receptors CB_1_ and CB_2_ and TRPV1

In the next step, we investigated whether the suppression of VCAM-1 and ICAM-1 by AEA is mediated by the activation of cannabinoid receptors or TRPV1. For this purpose, HCAEC were preincubated with receptor antagonists prior to coincubation with AEA and IL-1β or LPS. The protein expression of VCAM-1 and ICAM-1 was analyzed after 24 h.

**Figure 6:**
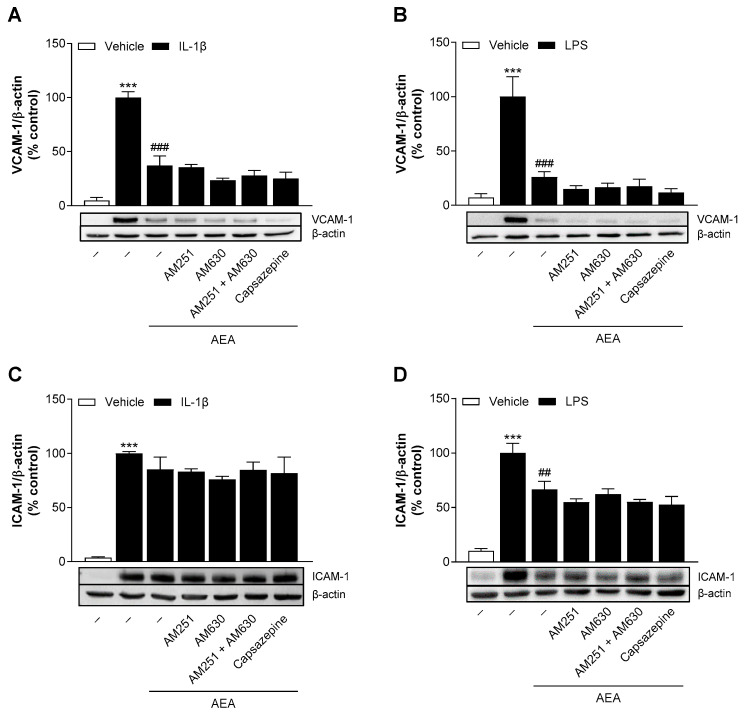
Analysis of the involvement of cannabinoid receptor targets in the effect of AEA on IL-1β- or LPS-induced VCAM-1 and ICAM-1 protein expression in HCAEC. Cells were preincubated with the respective receptor antagonist (1 µM) or vehicle for 1 h and then incubated for a further 24 h with 10 ng/mL IL-1β and AEA (10 µM) or vehicle. In the case of LPS, cells were preincubated for 1 h with the respective receptor antagonist or vehicle, then treated for 1 h with 10 µM AEA or vehicle, and finally coincubated for 24 h with 1 µg/mL LPS or vehicle. Protein expression was determined by Western blot analysis. Cells treated with IL-1β or LPS alone served as controls (100%). The values were normalized to β-actin and presented as means ± SEM of *n* = 3 (**A**,**C**), *n* = 4 (**B**) or *n* = 5 (**D**) independent experiments. *** *p* ≤ 0.001 vs. vehicle control; ## *p* ≤ 0.01, ### *p* ≤ 0.001 vs. IL-1β or LPS control; one-way ANOVA plus Bonferroni post hoc test.

Consistent with the above findings, stimulation with IL-1β or LPS alone resulted in a strong upregulation of VCAM-1 and ICAM-1 protein expression, approximately 14–21-fold and 10–27-fold above baseline values, respectively (Figure 6A,B,C,D). AEA significantly attenuated the induced VCAM-1 expression under IL-1β and LPS conditions by approximately 63% and 74%, respectively, compared to stimulated controls (Figure 6A,B). Treatment with antagonists targeting CB_1_ (AM251), CB_2_ (AM630) or TRPV1 (capsazepine) did not prevent AEA-induced VCAM-1 downregulation after IL-1β or LPS stimulation (Figure 6A,B). As a control, we also examined IL-1β-induced ICAM-1 expression. As already shown above (Figure 3E), no effect of AEA was observed in this case (Figure 6C). In LPS-stimulated endothelial cells, AEA-mediated ICAM-1 suppression by approximately 33% was also independent of the investigated cannabinoid receptors (Figure 6D).

In our previous study [16], we did not detect cytotoxic effects of the receptor antagonists alone on HCAEC. On the other hand, we observed an inhibitory effect of capsazepine on the induced VCAM-1 protein levels [16]. In the present study, capsazepine and the combination of AM251 and AM630 each reduced LPS-induced ICAM-1 expression in HCAEC by approximately 30% (Supplementary Figure S3).

### 3.9. AEA Has No Significant Effect on the Activation of the NF-κB Signal Transduction Cascade in HCAEC

With the aim of elucidating the molecular mechanisms underlying AEA-mediated VCAM-1 and ICAM-1 suppression, we have investigated several signaling pathways in the following. In our previous study [16], it was already determined through inhibitor experiments that the nuclear factor kappa-light-chain-enhancer of activated B cells (NF-κB) regulates the expression of both adhesion molecules in IL-1β- and LPS-stimulated HCAEC. Furthermore, the transcriptional regulation of VCAM-1 and ICAM-1 by NF-κB has been described in several other studies [35,36].

Although no profound effects on the expression of VCAM-1 and ICAM-1 could be demonstrated for 2-AG in this study, we decided to continue using this endocannabinoid as a comparator to AEA. We first examined the effects of AEA or 2-AG on the induced phosphorylation of the upstream NF-κB signal transduction cascade protein IκB kinase (IKKβ) and the inhibitor of NF-κB (IκBα) over time. For this purpose, HCAEC were treated with the test substances and stimulated with IL-1β or LPS. The induced phosphorylation of IKKβ and IκBα was analyzed using the Western blot technique.

**Figure 7:**
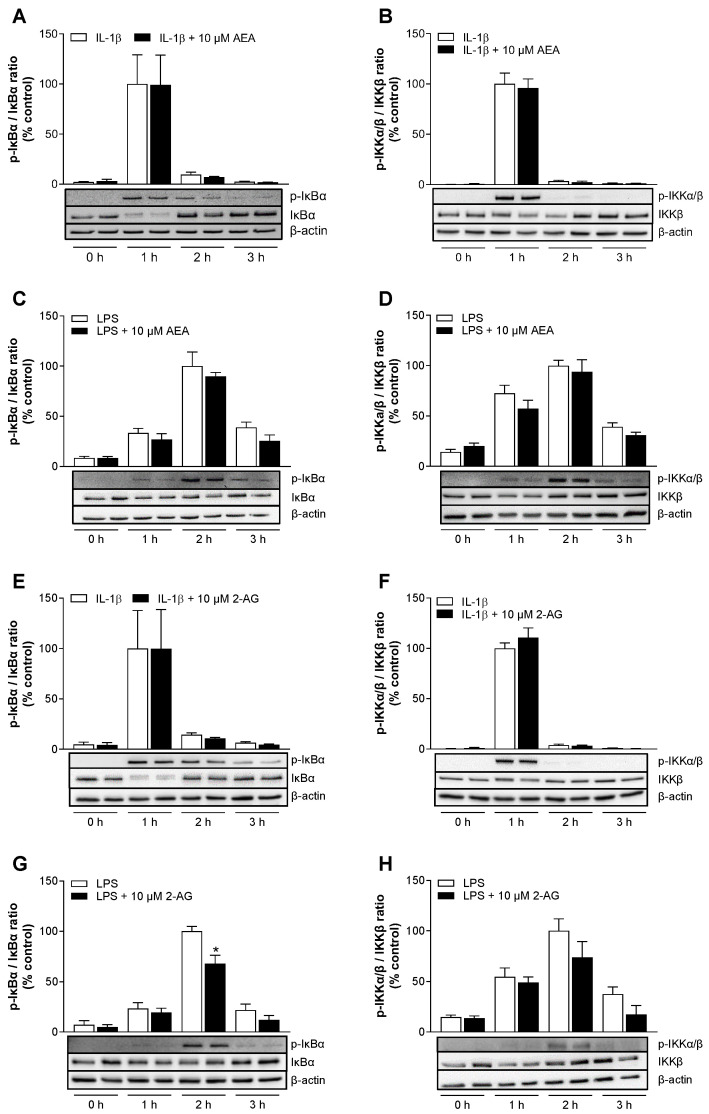
Impact of AEA (**A**–**D**) or 2-AG (**E**–**H**) on IL-1β- or LPS-induced phosphorylation of IKKα/β and IκBα in HCAEC. Cells were treated with 10 µM AEA or 10 µM 2-AG or vehicle and coincubated with 10 ng/mL IL-1β. For analysis of LPS-induced NF-κB activation, cells were preincubated with 10 µM AEA or 10 µM 2-AG or vehicle 1 h prior to addition of LPS (1 µg/mL). Protein samples were collected following stimulation for the indicated time points. Western blotting was performed for IKKβ, IκBα and its phosphorylated forms (p-IKKα/β, p-IκBα). Cells treated with IL-1β for 1 h or with LPS for 2 h served as controls (100%). Protein expression values were normalized to β-actin as equal loading control and represented as means ± SEM of *n* = 4 (**A**–**C**) or *n* = 3 (**D**–**H**) independent experiments. * *p* ≤ 0.05 vs. time-matched LPS control group; Student’s unpaired two-tailed t test.

As shown in Figure 7A,B,E,F, the strongest IL-1β-induced activation of IκBα and IKKβ was detected after 1 h. In LPS-stimulated cells, maximal IκBα and IKKβ phosphorylation occurred after 2 h (Figure 7C,D,G,H). AEA treatment showed no effect on IL-1β-induced NF-κB signaling pathway (Figure 7A,B), but slightly, though not significantly, decreased LPS-induced IκBα and IKKβ phosphorylation (Figure 7C,D). Similarly, 2-AG displayed no influence on IL-1β-induced IκBα and IKKβ activation, while IκBα phosphorylation by LPS was significantly suppressed by 32% at its maximum after 2 h (Figure 7G).

### 3.10. AEA Shows No Inhibitory Effect on the Phosphorylation of p38 MAPK

The p38 mitogen-activated protein kinase (MAPK) was next examined as another important signaling pathway that plays a central role in modulating the endothelial response to pro-inflammatory stimuli [37,38]. Here, previous inhibitor experiments by our group had shown that p38 MAPK mediates IL-1β- and LPS-induced VCAM-1 but not ICAM-1 expression in HCAEC [16].

The stimulation of the p38 MAPK signaling pathway by IL-1β and LPS was initially examined over time. A maximum p38 MAPK phosphorylation was detected after a 30 min exposure to IL-1β, with an about 3–4-fold increase compared to 0 h (Supplementary Figure S4A,B). A corresponding coincubation with AEA or 2-AG did not lead to any inhibition of p38 MAPK activation recorded after 15 min, 30 min or 60 min of stimulation with IL-1β (Supplementary Figure S4A,B).

Compared to IL-1β, it required a longer time for LPS stimulation to activate p38 MAPK, starting at 1 h with a 1.6-fold increase (Supplementary Figure S4C), so the exposure time to LPS was increased to up to 2 h in follow-up experiments (Supplementary Figure S4D,E). This confirmed that LPS-induced p38 MAPK phosphorylation reached a maximum after 1.5 h. Again, AEA did not prevent p38 MAPK phosphorylation when exposed to LPS at either time point (Supplementary Figure S4C,D). In contrast to AEA, 2-AG slightly, but significantly, reduced the maximum LPS-induced activation of p38 MAPK after 1.5 h (Supplementary Figure S4E) and caused an increase under both stimuli at 0 h (Supplementary Figure S4B,E).

### 3.11. AEA Significantly Inhibits Basal and IL-1β-Mediated Activation of Akt in HCAEC

**Figure 8:**
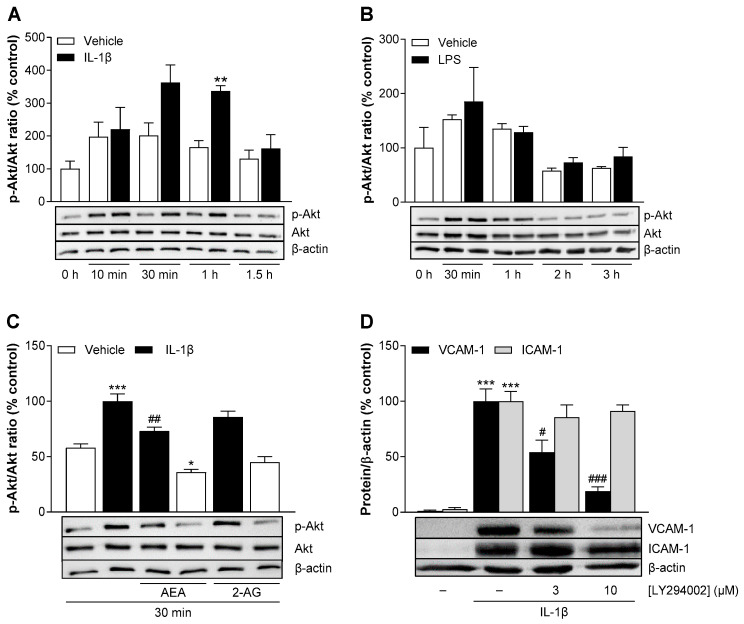
Time course of IL-1β- (**A**) or LPS (**B**)-induced Akt phosphorylation in HCAEC. Cells were incubated in the presence or absence of 10 ng/mL IL-1β or 1 µg/mL LPS or vehicle. Protein samples were collected following incubation for the indicated times. Western blot analysis was performed for Akt and its phosphorylated form (p-Akt). (**C**) Influence of AEA or 2-AG on basal and IL-1β-induced Akt phosphorylation. Here, cells were incubated with 10 µM AEA or 10 µM 2-AG or vehicle and 10 ng/mL IL-1β or vehicle for 30 min. (**D**) Effect of LY294002 (PI3K inhibitor) on IL-1β-induced VCAM-1 and ICAM-1 protein expression in HCAEC. Cells were preincubated with LY294002 at a concentration of 3 µM or 10 µM or vehicle for 1 h and then coincubated with 10 ng/mL IL-1β or vehicle for 24 h. Protein expression (**A**–**D**) was determined by Western blot analysis and normalized to β-actin as equal loading control. Vehicle-treated (**A**,**B**) or IL-1β-treated cells (**C**,**D**) served as control (set as 100%). Data are shown as means ± SEM of *n* = 3 (**A**,**B**,**D**) or *n* = 4 (**C**) independent experiments. ** *p* ≤ 0.01 vs. time-matched vehicle control; Student’s unpaired two-tailed t test (**A**) or * *p* ≤ 0.05, *** *p* ≤ 0.001, vs. vehicle control; # *p* ≤ 0.05, ## *p* ≤ 0.01, ### *p* ≤ 0.001 vs. stimulated control group; one-way ANOVA plus Bonferroni post hoc test (**C**,**D**).

In view of other studies that had shown a role for phosphatidylinositol 3-kinase (PI3K)/Akt in the regulation of adhesion molecules [39,40], we next examined this pathway as a potential target of AEA. To determine the time of maximum Akt activation, HCAEC were incubated in the presence or absence of IL-1β for up to 1.5 h. Akt phosphorylation was determined by Western blot analysis and then normalized to the unphosphorylated form, which is hereafter defined as activation. IL-1β increased Akt activation by about 1.8-fold after 30 min of incubation and maintained this stimulation up to 1 h. Thereafter, phosphorylation decreased sharply (Figure 8A). Based on the data from the NF-κB and p38 MAPK experiments, we expected a longer activation time by LPS and therefore chose incubation times of up to 3 h. However, Akt activation by LPS could not be detected at any time point (Figure 8B).

We next examined the effects of AEA and 2-AG on IL-1β-induced Akt phosphorylation at its peak time point. For this, HCAEC were incubated with AEA or 2-AG and IL-1β or its vehicle for 30 min. As a result, AEA significantly reduced basal and IL-1β-induced Akt phosphorylation (Figure 8C). 2-AG showed a non-significant reduction in Akt phosphorylation.

To determine the involvement of the PI3K/Akt pathway in the IL-1β-mediated VCAM-1 protein expression, HCAEC were preincubated with the PI3K/Akt inhibitor LY294002 for 1 h before stimulation with IL-1β. Changes in VCAM-1 and ICAM-1 protein expression were determined after 24 h. LY294002 showed a concentration-dependent downregulation of VCAM-1 by 46% at 3 µM and by 81% at 10 µM (Figure 8D). On the other hand, LY294002 caused only a slight, non-significant decrease in ICAM-1 expression of 9–14%. LY29002 elicited a cytotoxic effect on HCAEC at the higher concentration used (Supplementary Table S2), which, however, was quantitatively less than the massive VCAM-1 downregulation triggered by this concentration.

### 3.12. AEA Significantly Inhibits LPS-Induced Phosphorylation of STAT3 in HCAEC

**Figure 9:**
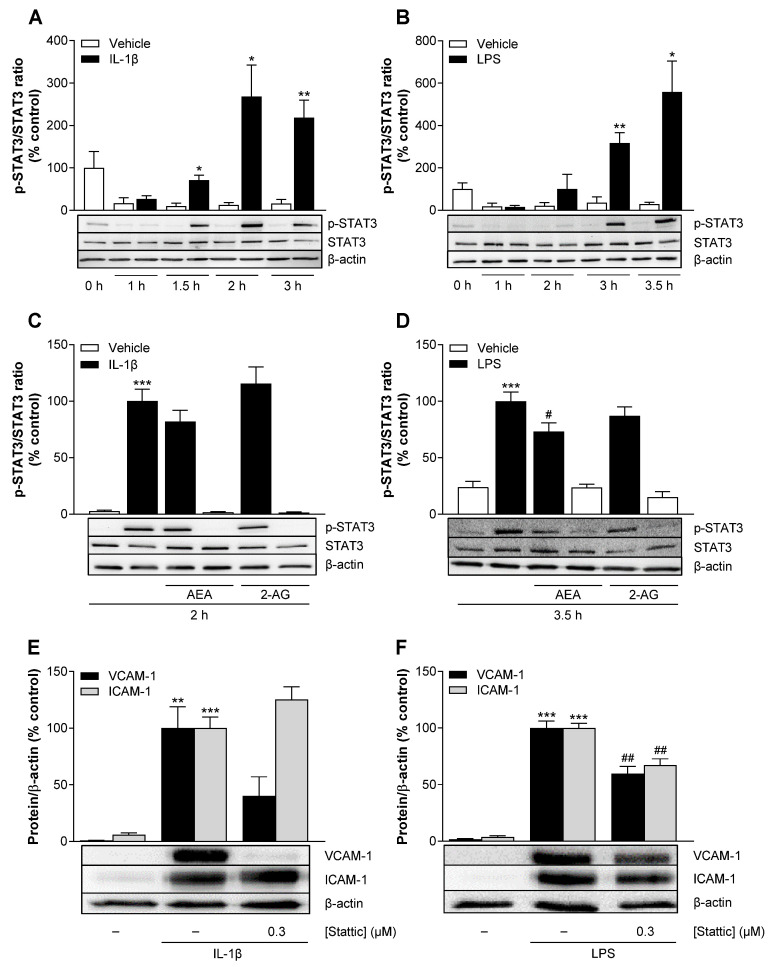
Time course of IL-1β- (**A**) or LPS (**B**)-induced STAT3 phosphorylation in HCAEC. Cells were incubated in the presence or absence of 10 ng/mL IL-1β or 1 µg/mL LPS or vehicle. Protein samples were collected following incubation for the indicated times. Western blot analysis was performed for STAT3 and its phosphorylated form (p-STAT3). Influence of AEA or 2-AG on basal and IL-1β- (**C**) or LPS (**D**)-induced STAT3 phosphorylation. Here, cells were incubated with 10 µM AEA or 10 µM 2-AG or vehicle and 10 ng/mL IL-1β or vehicle for 2 h. For experiments under LPS-induced conditions, cells were preincubated with 10 µM AEA or 10 µM 2-AG or vehicle for 1 h and then incubated for another 3.5 h after addition of 1 µg/mL LPS or vehicle. Effect of Stattic on IL-1β- (**E**) or LPS (**F**)-induced VCAM-1 and ICAM-1 protein expression in HCAEC. Cells were preincubated with 0.3 µM Stattic or vehicle for 1 h and then coincubated with 10 ng/mL IL-1β or 1 µg/mL LPS or vehicle for an additional 24 h. Protein expression was determined by Western blot analysis and normalized to β-actin as equal loading control. Cells treated with vehicle (**A**,**B**) or IL-1β (**C**,**E**) or LPS (**D**,**F**) alone served as control (set as 100%). Data are shown as means ± SEM of *n* = 3 (**A**,**B**,**E**,**F**), *n* = 5 (**C**) or *n* = 7 (**D**) independent experiments. * *p* ≤ 0.05, ** *p* ≤ 0.01 vs. time-matched vehicle control; Student’s unpaired two-tailed t test (**A**,**B**) or ** *p* ≤ 0.01, *** *p* ≤ 0.001 vs. vehicle control group; # *p* ≤ 0.05, ## *p* ≤ 0.01 vs. LPS control group; one-way ANOVA plus Bonferroni post hoc test (**C**–**F**).

Previous studies have shown that STAT3 plays a role in the regulation of VCAM-1 and ICAM-1 expression in endothelial cells [41,42]. To extend our analysis of molecular mechanisms, we therefore investigated a possible involvement of STAT3 activation in the expression of both adhesion molecules in HCAEC. Since STAT3 is a mediator acting further downstream in the signaling pathways, we extended the times for stimulation with IL-1β or LPS to monitor phosphorylation over time. Figure 9A,B shows significant STAT3 activation under IL-1β- and LPS-stimulated conditions. IL-1β-induced phosphorylation peaked at 2 h, while LPS reached the highest activation at 3.5 h. We chose these time points for the respective stimulants to examine the effects of AEA and 2-AG on STAT3 phosphorylation by immunoblotting.

Coincubation of AEA and IL-1β reduced the induced phosphorylation of STAT3 by 18%, albeit not significantly (Figure 9C). In contrast, 2-AG showed no effect on STAT3 activation under IL-1β conditions. On the other hand, AEA significantly attenuated LPS-induced STAT3 phosphorylation by 27%, whereas 2-AG only caused a slight reduction of 13% (Figure 9D).

In further experiments, Stattic, a STAT3 inhibitor, attenuated IL-1β-induced VCAM-1 but not ICAM-1 protein expression (Figure 9E). In contrast, in LPS-stimulated HCAEC, Stattic significantly decreased the expression of both adhesion molecules (Figure 9F). The concentration of Stattic used here has been shown to be non-toxic to HCAEC viability in both the WST-1 and crystal violet assays (Supplementary Table S2).

### 3.13. AEA Significantly Inhibits Phosphorylation of Src in IL-1β-Stimulated HCAEC

**Figure 10:**
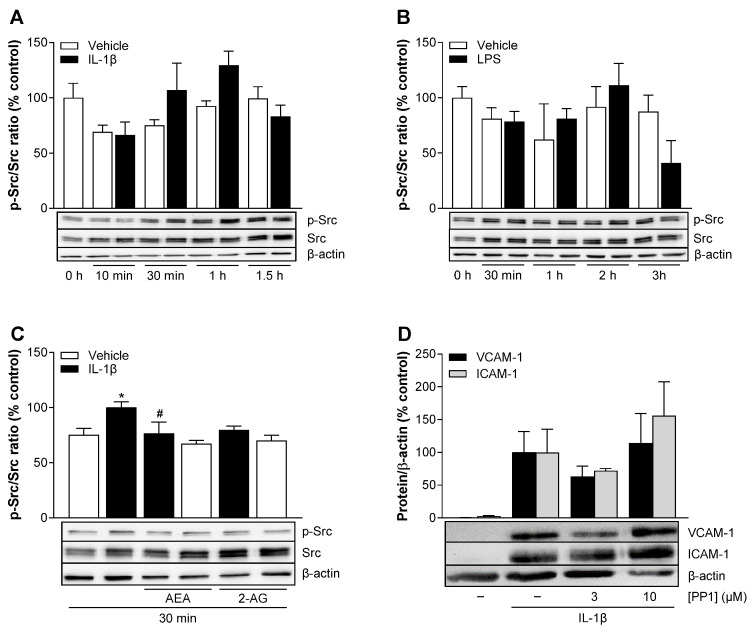
Time course of IL-1β- (**A**) or LPS (**B**)-induced Src phosphorylation in HCAEC. Cells were incubated in the presence or absence of 10 ng/mL IL-1β or 1 µg/mL LPS or vehicle. After stimulation, protein samples were collected at the indicated times. Western blot analysis was performed for Src and its phosphorylated form (p-Src). (**C**) Influence of AEA or 2-AG on basal and IL-1β-induced Src phosphorylation. Here, cells were incubated with 10 µM AEA or 10 µM 2-AG or a vehicle and 10 ng/mL IL-1β or a vehicle for 30 min. (**D**) Effect of PP1 (Src inhibitor) on IL-1β-induced VCAM-1 and ICAM-1 protein expression in HCAEC. Cells were preincubated with 3 µM or 10 µM PP1 or vehicle for 1 h and then coincubated with 10 ng/mL IL-1β or vehicle for 24 h. Protein expression was determined by Western blot analysis and normalized to β-actin as equal loading control. Cells treated with vehicle (**A**,**B**) or IL-1β (**C**,**D**) alone served as control (set as 100%). Data are shown as means ± SEM of *n* = 3 (**A**,**B**,**D**) or *n* = 5 (**C**) independent experiments. Statistical significance between time-matched groups was excluded using Student’s unpaired two-tailed t test (**A**,**B**). * *p* ≤ 0.05 vs. vehicle control; # *p* ≤ 0.05 vs. IL-1β control group; one-way ANOVA plus Bonferroni post hoc test (**C**,**D**).

The tyrosine kinase Src has also been linked to the expression of VCAM-1 and ICAM-1 [39,40]. To investigate the involvement of activated Src in the effects of AEA on the expression of VCAM-1 and ICAM-1, time course experiments were again performed first, and the phosphorylation of Src was determined by Western blot analysis. In IL-1β-treated HCAEC, Src phosphorylation increased by 43% after 30 min compared to the corresponding basal values and returned to the basal values after 1.5 h of incubation (Figure 10A). By contrast, Src activation was not detectable at any time point after the start of LPS incubation (Figure 10B).

Concomitant incubation of HCAEC with IL-1β and AEA or 2-AG was associated with a (significant) 23% and a (non-significant) 20% decrease in IL-1β-induced Src phosphorylation, respectively (Figure 10C). The VCAM-1 and ICAM-1 expression induced by IL-1β was downregulated in the presence of the Src inhibitor PP1 at a concentration of 3 µM (Figure 10D). However, an upregulation of VCAM-1 and ICAM-1 was observed when a higher concentration of PP1 (10 µM) was used. The results of the colorimetric WST-1 test and the crystal violet staining showed no significant cytotoxic effect of PP1 at a concentration of 3 µM but a strong decrease in metabolic activity and cell number at a concentration of 10 µM (Supplementary Table S2). The latter could be a cause of VCAM-1 and ICAM-1 upregulation as part of a stress-related response.

### 3.14. AEA Decreases IL-1β- and LPS-Induced Monocyte Adhesion to HCAEC

**Figure 11:**
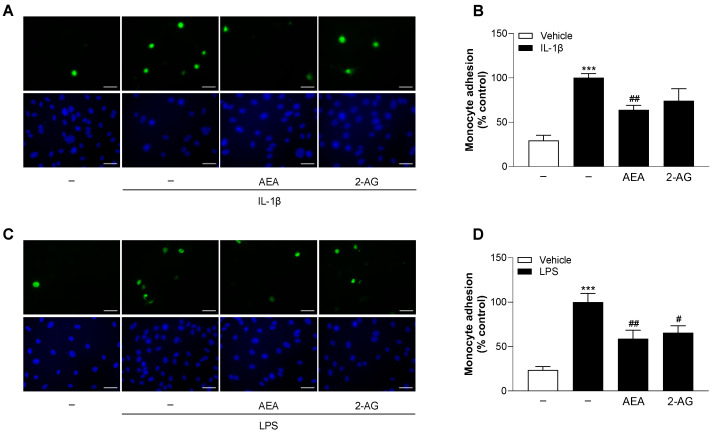
Effect of AEA and 2-AG on IL-1β- (**A**,**B**) or LPS (**C**,**D**)-induced monocyte adhesion to HCAEC. HCAEC were incubated with 10 µM AEA or 10 µM 2-AG or vehicle together with 10 ng/mL IL-1β or its vehicle for 24 h. For analysis under LPS conditions, cells were preincubated with AEA or 2-AG or vehicle 1 h prior coincubation with 1 µg/mL LPS or vehicle for 24 h. Thereafter, HCAEC were co-cultivated with calcein-AM-stained THP-1 cells for 30 min. Not adhered cells were removed by gentle washing. THP-1 cells adhering to endothelial cells (green) were detected by fluorescence microscopy. The nuclei of all cells are stained blue (Hoechst 33342), and the scale bar indicates 50 µm. Adhesion was calculated using the following formula: % adhesion = (number of calcein-AM-positive cells/number of Hoechst 33342-positive cells) × 100. IL-1β- (**B**) or LPS (**D**)-stimulated controls were defined as 100%. Data are represented as means ± SEM of *n* = 13 (7 independent experiments, (**A**,**B**)) or *n* = 11–12 (6 independent experiments, (**C**,**D**)). *** *p* ≤ 0.001 vs. corresponding vehicle; # *p* ≤ 0.05, ## *p* ≤ 0.01 vs. corresponding stimulated group; one-way ANOVA plus Bonferroni post hoc test.

In a further approach, we finally examined the functional impact of the influence of endocannabinoids on IL-1β- and LPS-induced endothelial expression of VCAM-1 and ICAM-1 on monocyte adhesion. For this purpose, HCAEC were treated with AEA or 2-AG in combination with IL-1β or LPS for 24 h and then incubated with calcein-AM-labeled THP-1 cells for 30 min. Fluorescence microscopy-based image analysis revealed a significant increase in monocyte adhesion to HCAEC by 3.4- or 4.2-fold after IL-1β or LPS treatment alone compared to vehicle-treated controls (Figure 11B,D). AEA significantly reduced the IL-1β- or LPS-induced adhesion of THP-1 monocytes to HCAEC by 36% and 41%, respectively. 2-AG also showed a reduction in IL-1β- and LPS-mediated monocyte adhesion of 26% (not significant) and 35% (significant), respectively.

### 3.15. siRNA-Mediated Silencing of IL-1β- and LPS-Induced Endothelial VCAM-1 or ICAM-1 Expression Significantly Reduces the Adhesion of THP-1 Cells to HCAEC

To definitively prove the functional significance of VCAM-1 and ICAM-1 in the adhesion of THP-1 cells to HCAEC in our experimental system, we used siRNA to knock down the expression of the adhesion molecules in corresponding experimental setups. To this end, HCAEC were treated with VCAM-1 siRNA, ICAM-1 siRNA or control siRNA, and monocyte adhesion experiments were performed as described above. To verify the knockdown, a Western blot analysis of VCAM-1 and ICAM-1 in treated HCAEC was performed in parallel with the adhesion experiments.

**Figure 12:**
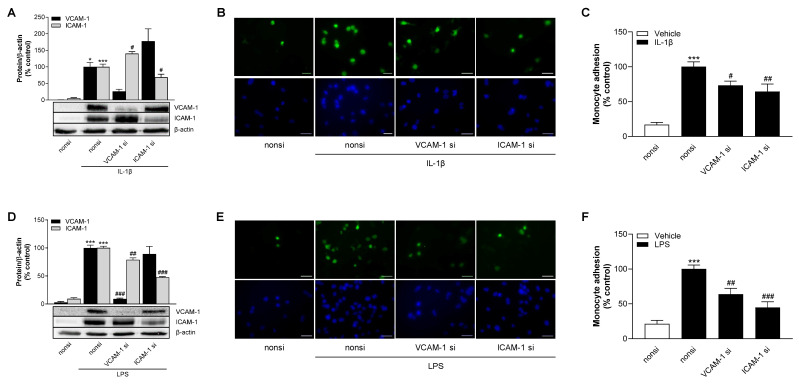
Effect of VCAM-1 and ICAM-1 siRNA transfection on IL-1β- (**A**–**C**) or LPS (**D**–**F**)-induced monocyte adhesion to HCAEC. HCAEC were seeded with a negative control siRNA (non-silencing siRNA, nonsi), VCAM-1 siRNA or ICAM-1 siRNA at 10 nM each and allowed to adhere for 24 h. Cells were then treated with 10 ng/mL IL-1β or 1 µg/mL LPS or vehicle for a further 24 h. (**A**,**D**) Knockdown efficiency of VCAM-1 and ICAM-1 were determined by Western blot analysis and normalized to β-actin. Cells treated with IL-1β or LPS and non siRNA served as controls (set as 100%). (**B**,**C**,**E**,**F**) Adhesion analysis of monocytes to endothelial cells was performed as described previously. Adhesion was calculated using the following formula: % adhesion = (number of calcein-AM-positive cells/number of Hoechst 33342-positive cells) × 100. IL-1β- (**A**,**B**) or LPS (**D**,**E**)-stimulated controls were defined as 100%. Data are shown as means ± SEM of *n* = 3 independent experiments (**A**,**D**), *n* = 8 (4 independent experiments (**B**,**C**)) or *n* = 6 (3 independent experiments (**E**,**F**)). * *p* ≤ 0.05, *** *p* ≤ 0.001 vs. corresponding vehicle; # *p* ≤ 0.05, ## *p* ≤ 0.01, ### *p* ≤ 0.001 vs. corresponding stimulated control; one-way ANOVA plus Bonferroni post hoc test.

As shown in Figure 12A,D, VCAM-1 siRNA reduced VCAM-1 protein expression to 26% and 9% of the corresponding 100% control values of IL-1β and LPS controls, respectively, transfected with non-silencing siRNA. Likewise, a pronounced reduction in ICAM-1 levels was achieved by ICAM-1 siRNA (Figure 12A,D). Here, decreases to 69% and 48% of the control value were observed under IL-1β- and LPS-stimulated conditions, respectively. It is noteworthy that the repression of IL-1β-induced VCAM-1 protein expression led to a counterregulation of ICAM-1 expression, while silencing of ICAM-1 caused an increase in VCAM-1 expression (Figure 12A). This phenomenon did not occur in LPS-stimulated cells (Figure 12D). Here, downregulation of LPS-induced VCAM-1 protein expression led to a slight decrease in ICAM-1 expression (by 21%, significant), whereas the knockdown of ICAM-1 resulted in a slight reduction in VCAM-1 (by 11%, not significant).

THP-1 adhesion to endothelial cells was again assessed by fluorescence-based image analysis. Here, treatment with VCAM-1 or ICAM-1 siRNA significantly suppressed IL-1β-mediated monocyte adhesion to HCAEC by 27% and 35%, respectively (Figure 12B,C). Under LPS conditions, treatment with VCAM-1 or ICAM-1 siRNA also resulted in a significant decrease in THP-1 adhesion by 36% and 55%, respectively (Figure 12E,F).

The effect of siRNA on the viability of HCAEC was evaluated using the WST-1 assay and crystal violet staining (Supplementary Table S3). At the concentrations used, VCAM-1 siRNA and ICAM-1 siRNA showed no significant change in cell number. With VCAM-1 siRNA, slight decreases in metabolic activity were observed, but these were considerably less than the percentage of VCAM-1 knockdown mediated by VCAM-1 siRNA.

## 4. Discussion

Focusing on potential protective effects of the endocannabinoid AEA in coronary artery cells, the present study demonstrates that AEA impairs the migration of PDGF-stimulated HCASMC and also leads to a reduced expression of adhesion molecules in HCAEC stimulated with various inflammatory mediators, thus inhibiting endothelial adhesion of monocytes.

In the case of PDGF-stimulated HCASMC, AEA caused a significant inhibition of migration without affecting proliferation. 2-AG, on the other hand, did not inhibit either PDGF-induced proliferation or migration. The antimigratory effect demonstrated for AEA appears significant in view of the fact that excessive migration of VSMC can be demonstrated in atherosclerotic processes and after coronary stenting. In the latter case, VSMC proliferation and migration is the most common cause of restenosis in the stent, so that the search for drugs with a corresponding inhibitory effect continues to represent a pharmacotherapeutic challenge (for review, see [21]). Following recently published findings with CBD and THCV [16], AEA has now been described as another cannabinoid that leads to a corresponding inhibition of HCASMC migration in non-toxic concentrations. The antimigratory property of AEA was independent of the activation of the major AEA receptor targets CB_1_, CB_2_ and TRPV1. With regard to the use of other cannabinoids, it should be noted that a previous study has shown that the CB_1_ receptor antagonist rimonabant (SR141716) attenuates the proliferation and migration of HCASMC at concentrations of 1 µM and above [14]. Meanwhile, however, a number of CB_1_-independent effects have been compiled for rimonabant [43], so that, in our opinion, the aforementioned earlier findings on migration should be re-evaluated using further pharmacological and genetic tools. In the present study, another CB_1_ antagonist, AM251, which differs from rimonabant by a halogen substituent, was used to investigate the receptor dependence of AEA. Here, AM251 at a concentration of 1 µM did not lead to a decrease in PDGF-induced migration.

With regard to the HCAEC used by us, a previous study has raised concerns about the toxicity of AEA in these cells. Indeed, in this investigation, AEA was shown to induce apoptosis and necrosis at concentrations of 5 and 10 µM [10]. Thereby, corresponding cytotoxicities were significantly attenuated in the presence of CB_1_ and CB_2_ receptor antagonists as well as inhibitors of p38 and c-Jun NH_2_-terminal protein kinase (JNK) MAPK. In light of this, two different cytotoxicity assays were carried out at the beginning of our study, neither of which registered any significant loss of viability for AEA. In the case of 2-AG, at the highest tested concentration of 10 µM, we were able to register moderate cell toxicity in the crystal violet assay but not in the WST-1 test. Although no comparative data are available for exogenous 2-AG, a recently published study showed that 24 h treatment of HCAEC with JZL184, an inhibitor of 2-AG-degrading monoacylglycerol lipase, was associated with reduced cell viability [15]. However, JZL184 did not decrease the viability of confluent HCAEC, suggesting inhibition of proliferation as the cause of decreased viability rather than induction of apoptosis or cell death.

A key finding of this work is the demonstration of VCAM-1 inhibition by AEA in the presence of two different inflammatory stimuli and the subsequent inhibition of monocyte adhesion. In addition, a significant reduction in ICAM-1 expression by AEA was registered selectively under LPS conditions. The effects mentioned were also detectable at the transcriptional level and occurred independently of the activation of CB_1_, CB_2_ and TRPV1. A selective inhibition of endothelial VCAM-1, but not ICAM-1, is frequently found in the literature for various test substances [44,45,46,47] and was recently also described for CBD and THCV in HCAEC stimulated with IL-1β and, in contrast to our findings presented here with AEA, also with LPS [16]. Since the endothelial VCAM-1 expression is known to be sensitive to the cellular redox environment [48], an antioxidant effect was attributed to the corresponding test substances in some of the studies cited above.

Inhibition of VCAM-1 expression by AEA has also been previously demonstrated in endothelial cells. For example, AEA from a concentration of 5 µM caused a significant CB_1_ receptor-dependent inhibition of VCAM-1 expression in mouse brain endothelial cells infected with Theiler virus (TMEV), the functional significance of which could be confirmed by leukocyte transmigration assay [49]. The VCAM-1 inhibition shown in the cerebral vasculature was interpreted as a new mechanism to explain the therapeutic effect of increased AEA tone in neuroinflammatory diseases such as multiple sclerosis. In another study, AEA and 2-AG, each at a concentration of 10 µM, caused a partial but significant inhibition of TNF-α-induced VCAM-1 expression in human aortic endothelial cells [50]. Finally, and in agreement with our findings using IL-1β and LPS as inflammatory inducers, AEA, tested at a concentration of 10 µM, was found to inhibit TNF-α-induced VCAM-1 expression in HCAEC [8]. However, in contrast to our results under IL-1β stimulation of HCAEC, additional inhibition of ICAM-1 formation by AEA was observed in the presence of TNF-α in the cited work. Furthermore, the authors described the inhibition of TNF-α-induced VCAM-1 and ICAM-1 expression and monocyte adhesion by AEA as CB_1_ and CB_2_ receptor-dependent events. Conversely, in our hands, the inhibitory effect of AEA on VCAM-1 expression triggered by IL-1β or LPS was not reversed by CB_1_ and CB_2_ receptor antagonists. The causes of these divergent mechanisms of action are unclear and require further clarification. However, IL-1β and TNF-α are known to influence the inflammatory phenotype differently, as demonstrated in brain microvascular endothelial cells. Here, TNF-α induced a significantly stronger cell surface expression of ICAM-1 and VCAM-1 than IL-1β [51]. Likewise, in human microvascular endothelial cells (HMEC-1a), TNF-α proved to be a stronger inducer of VCAM-1 expression compared to IL-1β [52].

While ICAM-1 and VCAM-1 showed a strong induction in response to IL-1β and LPS, E-selectin was induced to a much lesser extent by IL-1β and not at all under LPS conditions at 24 h. Consistent with these findings, the E-selectin expression profile has been described by a rapid increase compared to the other adhesion molecules and a higher turnover rate [53]. In a time-course experiment performed in human umbilical vein endothelial cells (HUVEC), stimulation with LPS accordingly resulted in an induction of E-selectin within 1–2 h, peaking at 4–6 h, and gradually returning to baseline at 24 h [54]. In accordance with its kinetics, E-selectin is responsible for the first steps of the adhesion cascade, such as rolling and arrest of monocytes (for review, see [55,56]). ICAM-1 and VCAM-1, on the other hand, are mainly responsible for adhesion and transmigration and therefore most relevant for the findings of the THP-1 adhesion experiments performed after 24 h of HCAEC incubation.

Regarding the signal transduction of AEA-mediated inhibition of VCAM-1 formation, we could not demonstrate involvement of the NF-κB signaling pathway typical for induction of VCAM-1 expression (for review, see [57]) nor p38 MAPK acting as an upstream regulator of NF-κB [58]. These results were surprising because in a previous study from our laboratory, inhibition of NF-κB was associated with a reduction in VCAM-1 and ICAM-1 protein in IL-1β- and LPS-stimulated HCAEC, and the phytocannabinoids CBD and THCV led to a significant reduction in LPS-induced phosphorylation of IKKβ and IκBα [16]. In addition, inhibitory effects on the NF-κB signaling pathway have been repeatedly described for AEA [8,59,60]. However, in a thorough analysis of two components of this pathway, IKKα/β and IκBα, AEA showed no significant effect. Interestingly, and unexpectedly due to its rather weak effect on VCAM-1 and ICAM-1 expression, 2-AG significantly suppressed LPS-induced IκBα phosphorylation, which should lead to further analysis of the effect of this endocannabinoid on other NF-κB-sensitive inflammatory mediators in the future.

Of the kinases (Akt, Src) and transcription factors (STAT3) subsequently investigated, activation by both stimuli, i.e., IL-1β and LPS, could only be demonstrated for STAT3. In the latter context, a significant inhibition of the LPS-mediated increase in STAT3 phosphorylation was found for AEA but not for 2-AG. Furthermore, the STAT3 inhibitor Stattic reduced the LPS-induced expression of ICAM-1 and VCAM-1 in the HCAEC we used, which is consistent with the findings of another group obtained in HUVEC [41]. Although IL-1β-induced VCAM-1 expression was also inhibited by Stattic in our hands, AEA showed no significant effect on IL-1β-induced STAT3 phosphorylation. Regardless of this, the inhibitor experiments conducted with Stattic show that STAT3 also plays a role in the IL-1β-induced upregulation of VCAM-1 expression in HCAEC. An involvement of the STAT3 pathway has otherwise also been described for the induction of VCAM-1 and ICAM-1 by IL-6 in human aortic endothelial cells [42] or by TNF-α in HUVEC [61].

In the case of the PI3K/Akt pathway, significant Akt phosphorylation could only be demonstrated in the presence of IL-1β, which in turn was significantly suppressed by AEA but not by 2-AG. The importance of this target within the inhibitory effect of AEA on IL-1β-induced VCAM-1 expression is also supported by the finding that LY294002, a PI3K/Akt inhibitor, selectively inhibited IL-1β-induced VCAM-1 expression and left the corresponding ICAM-1 induction unaffected. In fact, the PI3K/Akt signaling pathway, which is crucial for the AEA effect under IL-1β stimulation, is well known as a mediator of VCAM-1 induction. Thus, in human cardiac fibroblasts, the PI3K/Akt cascade is involved in TNF-α-induced VCAM-1 expression [62]. Further, in a BRAF-mutated papillary thyroid cancer cell line (BCPAP), vemurafenib, an inhibitor of mutant BRAF activity, was shown to upregulate VCAM-1 expression, which was reduced by the administration of the Akt inhibitor MK2206 [63]. In addition, PI3K and Akt inhibitors caused a massive reduction in connective tissue growth factor (CTGF)-induced VCAM-1 expression in osteosarcoma cells [64]. Finally, phosphatase and tensin homolog deleted on chromosome 10 (PTEN), a strong negative regulator of the PI3K/Akt signaling pathway, selectively inhibited the expression of VCAM-1 but not ICAM-1 by modulating the PI3K/Akt/GSK-3β/GATA-6 signaling cascade in TNF-α-treated HUVEC [65]. To our knowledge, AEA-mediated VCAM-1 regulation by Akt has not yet been described in endothelial cells. However, other studies have reported inhibitory effects of AEA on Akt phosphorylation in a different context. In human chondrocytes, AEA showed interference with the Akt signaling pathway by inhibiting Akt phosphorylation [66]. Furthermore, AEA inhibited proliferation of human endometrial stromal cells by reducing Akt phosphorylation [67].

In the present study, AEA also significantly alleviated IL-1β-induced Src phosphorylation. Interestingly, there was a temporal correlation in IL-1β-induced Akt and Src phosphorylation, which could indicate that both targets could interfere with each other. In line with this assumption, earlier studies described that the IL-18-induced VCAM-1 protein expression in rheumatoid arthritis synovial fibroblasts and the TNF-α-induced VCAM-1 expression in human cardiac fibroblasts [62] are controlled by the Src/PI3K/Akt signaling pathway [39]. On the other hand, our results show that the Src inhibitor PP1, in contrast to the VCAM-1-selective effect of the PI3K/Akt inhibitor LY294002, inhibits both the IL-1β-induced expression of VCAM-1 and ICAM-1, which in turn argues against a sequential interaction of the two kinases. Overall, however, this question should be clarified in future studies.

With reference to the adhesion assay we used, post-transcriptional knockdown of VCAM-1 and ICAM-1 clearly demonstrated the involvement of both proteins in the adhesion of THP-1 monocytes to IL-1β- and LPS-stimulated HCAEC. Although the efficiency of knockdown by VCAM-1 siRNA was even higher, ICAM-1 siRNA proved to be more potent in suppressing both IL-1β- and LPS-induced monocyte adhesion. The comparatively higher contribution of ICAM-1 in the interaction between HCAEC and monocytes is also supported by experiments of other authors. Here, adhesion of U937 monocytes to HCAEC triggered by carbamylated low-density lipoprotein (LDL), the most abundant modified LDL isoform in human blood, was reduced by silencing LDL-induced ICAM-1 induction using siRNA or inhibition by neutralizing antibodies. On the other hand, post-transcriptional knockdown or neutralization of VCAM-1 showed no effect, but led to increased suppression of monocyte adhesion when ICAM-1 was inhibited at the same time [68].

Interestingly, 2-AG also proved to be an inhibitor of adhesion here, albeit to a somewhat lesser extent than AEA. These results were surprising, as 2-AG showed a comparatively small effect on the protein levels of both adhesion molecules. On the other hand, the non-significant inhibition of VCAM-1 expression by the relevant 2-AG concentration of 10 µM was nevertheless as high as 26% (IL-1β stimulation), which was obviously sufficient to reduce adhesion. Under LPS conditions, the significant anti-adhesion effect mediated by 2-AG was associated with a non-significant 31% suppression of VCAM-1 and a significant 18% reduction in ICAM-1. Interestingly, in another study, incubation of HCAEC with 2-AG at concentrations of 1 and 10 µM, without the presence of an inflammatory stimulus, promoted the adhesion of THP-1 monocytes, but did not lead to a concomitant change in the adhesion molecules E-selectin, ICAM-1 and VCAM-1 [15].

Effects independent of CB_1_, CB_2_ or TRPV1 activation, as in our case for the inhibition of HCASMC migration and VCAM-1 and ICAM-1 expression in HCAEC, have been repeatedly described for AEA and other cannabinoids. For example, AEA has been shown to inhibit TNF-α-induced NF-κB activation independently of the membrane receptors mentioned by directly inhibiting IKKβ and, to a lesser extent, the IKKα subunits of the IκB kinase complex [59]. Moreover, cannabinoid receptor-independent inhibition of VCAM-1 and ICAM-1 expression is not new and has previously been described in astrocytoma cells using the synthetic cannabinoid R(+)WIN 55.212-2, where inhibition of transactivation of NF-κB was shown to be the underlying mechanism [69]. Other cannabinoid receptor-independent effects reported specifically for AEA include induction of oxidative stress or endoplasmic reticulum stress apoptosis in non-melanoma skin cancer cells [70], induction of necrosis in hepatic stellate cells [71], noncompetitive inhibition on 5-HT_3_ receptor-mediated responses [72] or inhibition of α7 nicotinic acetylcholine receptors [73]. In a recently published review, specific molecular mechanisms were proposed for the interaction of the arachidonic acid derivative AEA with membrane lipids and in particular with the binding partner cholesterol and ceramide, which could explain the corresponding receptor-independent effects of this endocannabinoid [5]. Clearly, in the case of the parameters we examined, further analyses would provide the relevant information.

**Figure 13:**
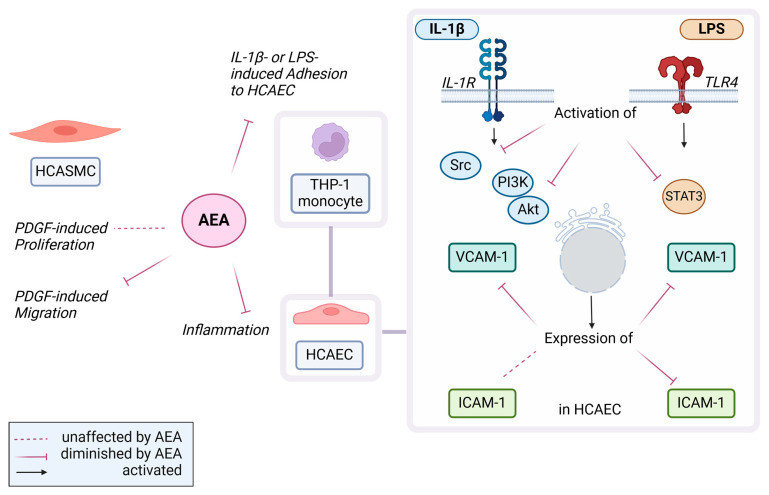
A schematic summary of the effects observed by AEA in this study. In PDGF-treated HCASMC, AEA reduced migration without affecting proliferation. In HCAEC, AEA inhibited IL-1β-induced VCAM-1 expression by interfering with PI3K/Akt and Src signaling pathways. Upon LPS stimulation, AEA inhibited VCAM-1 and ICAM-1 expression in HCAEC, which was preceded by inhibition of the activation of the transcription factor STAT3. Finally, an AEA-mediated inhibition of IL-1β- and LPS-induced adhesion of monocytes to HCAEC was demonstrated on a functional level. Common abbreviations used have been explained in the manuscript when first mentioned. Created with BioRender.com.

In summary, the present study demonstrated various protective effects of AEA on coronary artery cells (Figure 13). These range from an antimigratory effect on atherosclerosis- and restenosis-promoting HCASMC in the presence of increased growth factor concentrations to anti-inflammatory effects at the gene expression level, which ultimately led to reduced adhesion of atherosclerosis-promoting monocytes to HCAEC. Against this background and also in view of contradictory literature data on the role of the endocannabinoid system in direct effects on the coronary arteries and the myocardium, corresponding studies should be consistently continued.

## Data Availability

Data are available upon reasonable request from the first author.

## Supplementary Materials

The following supporting information can be downloaded at: https://www.mdpi.com/article/10.3390/cells13242108/s1, Supplementary Figure S1: Effect of AEA (A,B) and 2-AG (C,D) on metabolic activity and cell number of HCASMC; Supplementary Figure S2: Effect of AEA (A,B) and 2-AG (C,D) on metabolic activity and cell number of HCAEC; Supplementary Figure S3: Influence of antagonists at cannabinoid receptors or TRPV1 on LPS-induced ICAM-1 protein expression in HCAEC; Supplementary Figure S4: Impact of AEA (A,C,D) or 2-AG (B,E) on IL-1β- or LPS-induced phosphorylation of p38 MAPK in HCAEC; Supplementary Table S1: Effect of antagonists to cannabinoid receptors and TRPV1 on metabolic activity and cell number of HCASMC under PDGF-stimulated conditions; Supplementary Table S2: Effect of LY294002 (PI3K/Akt inhibitor), Stattic (STAT3 inhibitor) or PP1 (Src inhibitor) on metabolic activity and cell number of HCAEC under basal, IL-1β- or LPS-stimulated conditions; Supplementary Table S3: Effect of siRNA treatment on metabolic activity and cell number of HCAEC under IL-1β- or LPS-stimulated conditions.
