# Supplementary material for: Anandamide Inhibits Vascular Smooth Muscle Migration, Endothelial Adhesion Protein Expression and Monocyte Adhesion of Human Coronary Artery Cells
Source: Cells. 2024 Dec 19;13(24):2108. doi: 10.3390/cells13242108 (PMC11727187; doi:10.3390/cells13242108)
Supplement: Supplementary file 1 [file cells-13-02108-s001.zip › cells-3302385-supplementary.pdf]

Supplementary Materials

Anandamide Inhibits Vascular Smooth Muscle Migration, Endothelial Adhesion Protein Expression and Monocyte Adhesion of Human Coronary Artery Cells

Elane Blessing [1], Elisa Teichmann [1] and Burkhard Hinz [1,*]

[1] Institute of Pharmacology and Toxicology, Rostock University Medical Center, Schillingallee 70, 18057 Rostock, Germany; blessingelane@aol.de (E.B.)
* Correspondence: burkhard.hinz@med.uni-rostock.de

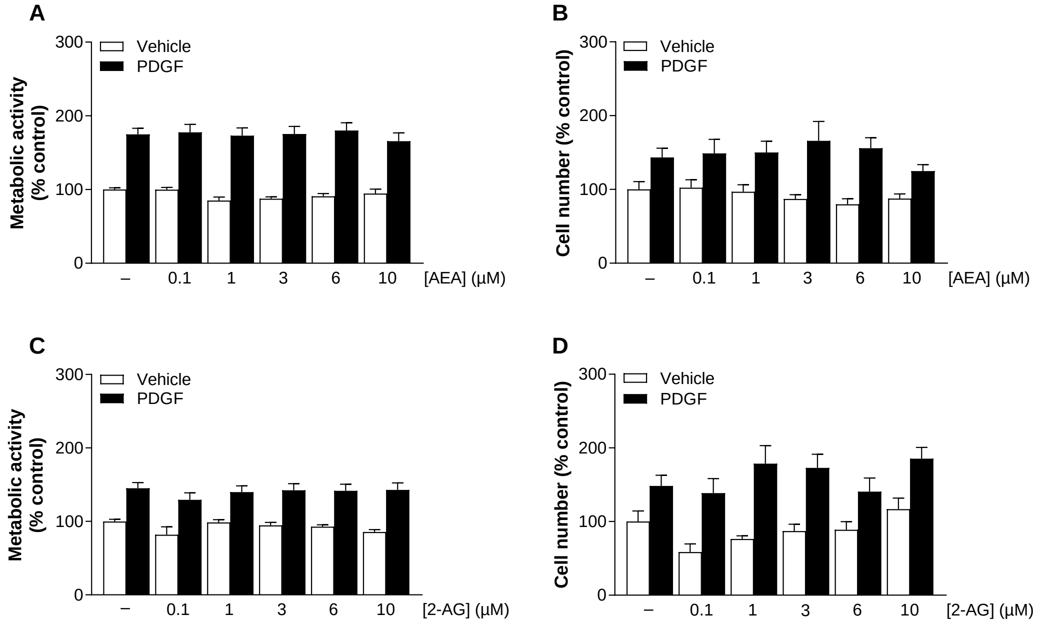

Supplementary Figure S1: Effect of AEA (**A**,**B**) and 2-AG (**C**,**D**) on metabolic activity and cell number of HCASMC. HCASMC were incubated with 25 ng/mL PDGF or its vehicle together with increasing concentrations of AEA or 2-AG or its vehicle. After 6 days of incubation (144 h), metabolic activity was analyzed using the WST-1 assay (**A**,**C**) and cell number was determined by crystal violet staining (**B**,**D**). Data are means ± SEM of n = 12 (4 independent experiments, (**A**)) or n = 9 (3 independent experiments, (**B**–**D**)). Significant effects of AEA and 2-AG under basal and PDGF-stimulating conditions were excluded by one-way ANOVA plus Dunnett post hoc tests.

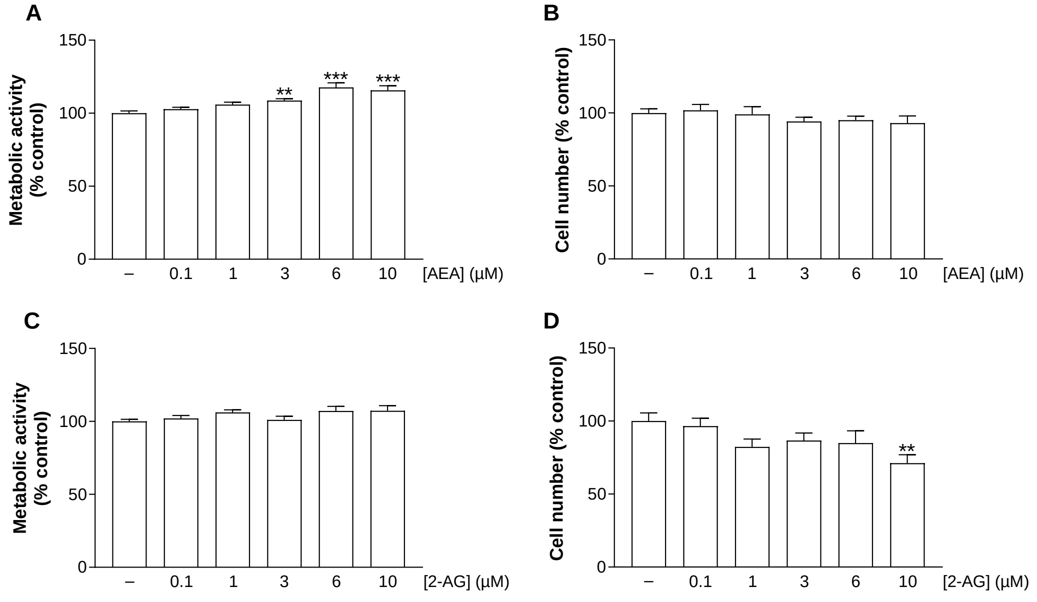

Supplementary Figure S2: Effect of AEA (**A**,**B**) and 2-AG (**C**,**D**) on metabolic activity and cell number of HCAEC. HCAEC were incubated with increasing concentrations of AEA or 2-AG or with vehicle for 24 h. Thereafter, metabolic activity was determined by WST-1 assay (**A**,**C**) and cell number by crystal violet staining (**B**,**D**). Vehicle-treated cells were used as controls (100%). Data are presented as means ± SEM of n = 16 (4 independent experiments, (**A**,**B**)) or n = 11–12 (3 independent experiments, (**C**,**D**)). ** $p \leq 0.01$, *** $p \leq 0.001$ vs. vehicle control; one-way ANOVA plus Dunnett post hoc test.

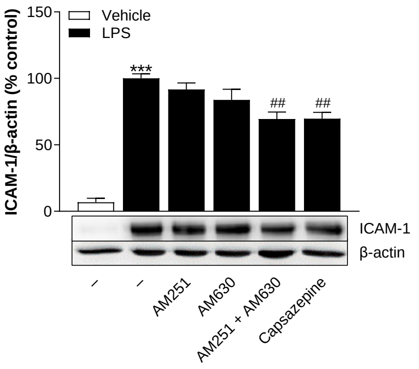

Supplementary Figure S3: Influence of antagonists at cannabinoid receptors or TRPV1 on LPS-induced ICAM-1 protein expression in HCAEC. Cells were preincubated with antagonists of the cannabinoid receptors CB_1 (AM251) and CB_2 (AM630) or TRPV1 (capsazepine), each at a concentration of 1 μM, or with vehicle for 1 h and then coincubated with 1 μg/mL LPS or vehicle for another 24 h. Protein expression was determined by Western blot analysis and normalized to β-actin. Cells stimulated with LPS alone were set 100%. Data are presented as means ± SEM of $n = 3$ independent experiments. *** $p \leq 0.001$ vs. vehicle control; ## $p \leq 0.01$ vs. LPS control; one-way ANOVA plus Bonferroni post hoc test.

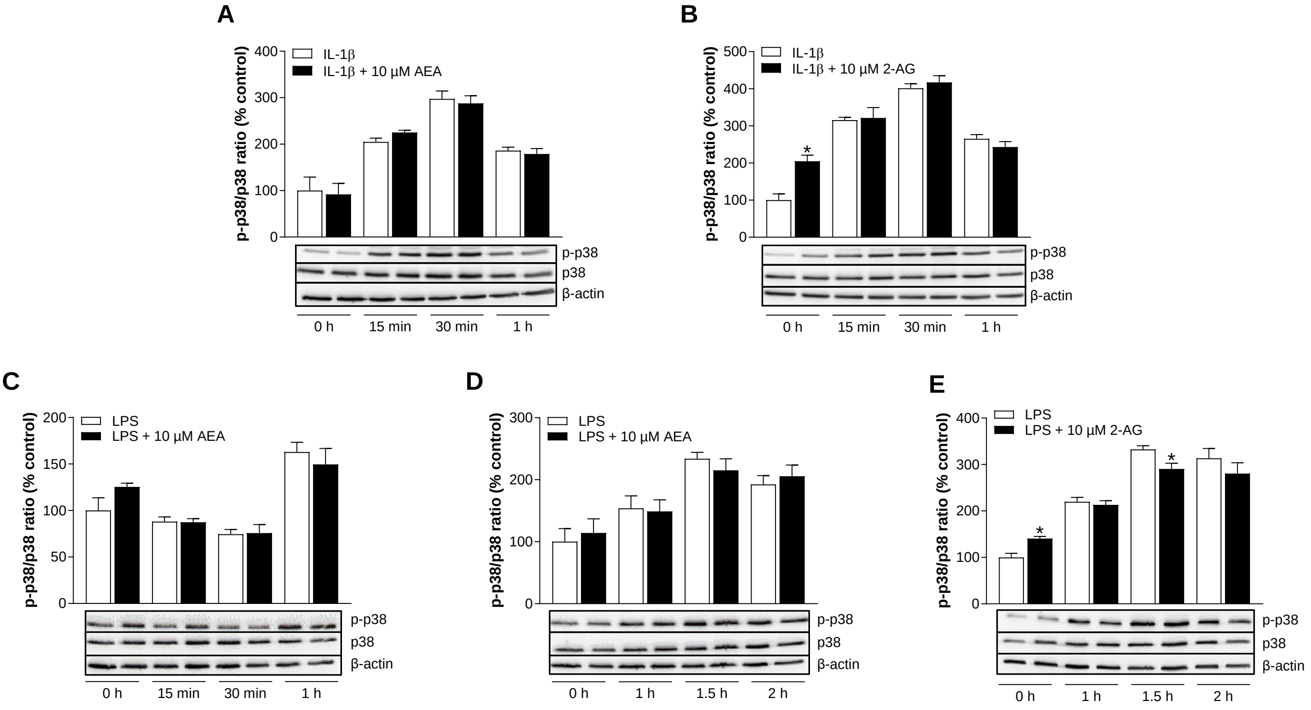

Supplementary Figure S4: Impact of AEA (**A**,**C**,**D**) or 2-AG (**B**,**E**) on IL-1β- or LPS-induced phosphorylation of p38 MAPK in HCAEC. Cells were treated with 10 μM AEA or 10 μM 2-AG or vehicle and coincubated with 10 ng/mL IL-1β. For analysis of LPS-induced p38 MAPK activation, cells were preincubated with 10 μM AEA or 10 μM 2-AG or vehicle 1 h prior to addition of LPS (1 μg/mL). Protein samples were collected following stimulation for the indicated times. Western blotting was performed for p38 MAPK and its phosphorylated form (p-p38). Protein expression values were normalized to β-actin as equal loading control. Cells stimulated with LPS alone (0 h) were set 100%. Data are presented as means ± SEM of $n = 3$ independent experiments. * $p \leq 0.05$ vs. time-matched vehicle control; Student's unpaired two-tailed t test.

Supplementary Table S1: Effect of antagonists to cannabinoid receptors and TRPV1 on metabolic activity and cell number of HCASMC under PDGF-stimulated conditions. HCASMC were maintained in serum-reduced medium (0.5% FCS) for 24 h. Cells were then preincubated with antagonists of the cannabinoid receptors CB_1 (AM251) and CB_2 (AM630) or TRPV1 (capsazepine), each at a concentration of 1 μM, or with a vehicle for 1 h, followed by the addition of 25 ng/mL PDGF or vehicle and 10 μM AEA or vehicle and continuing the incubation for another 24 h. Subsequently, metabolic activity was analyzed by WST-1 assay and cell number by crystal violet staining. Cells treated with the vehicle were used as control (set as 100%). Data are presented as means ± SEM of n = 6 (3 independent experiments). Statistically significant effects of PDGF, AEA, and receptor antagonists were excluded using a one-way ANOVA plus Bonferroni post hoc test.

Treatment group	Metabolic activity (%)	Cell number (%)
Vehicle	100.0 ± 12.5	100.0 ± 8.8
PDGF	150.4 ± 18.1	128.4 ± 24.3
AEA + PDGF	140.9 ± 12.2	130.0 ± 14.4
AM251 + AEA + PDGF	154.8 ± 15.3	129.0 ± 13.4
AM630 + AEA + PDGF	158.9 ± 22.0	130.9 ± 12.7
Capsazepine + AEA + PDGF	159.3 ± 15.9	106.9 ± 15.1
AM251 + PDGF	144.3 ± 11.2	124.8 ± 23.0
AM630 + PDGF	146.8 ± 19.4	127.6 ± 19.8
Capsazepine + PDGF	160.8 ± 13.5	175.2 ± 36.6

Supplementary Table S2: Effect of LY294002 (PI3K/Akt inhibitor), Stattic (STAT3 inhibitor) or PP1 (Src inhibitor) on metabolic activity and cell number of HCAEC under basal, IL-1β- or LPS-stimulated conditions. HCAEC were preincubated with the indicated inhibitors or vehicle for 1 h, followed by the addition of 10 ng/mL IL-1β, 1 μg/mL LPS or vehicle and continuing the incubation for another 24 h. Subsequently, metabolic activity was analyzed by WST-1 assay and cell number by crystal violet staining. Cells treated with vehicle were used as control (set as 100%). Data are presented as means ± SEM of n = 8–9 (3 independent experiments). ** $p \leq 0.01$ vs. vehicle control; # $p \leq 0.05$, ## $p \leq 0.01$, ### $p \leq 0.001$ vs. respective stimulated control; one-way ANOVA plus Bonferroni post hoc test.

Treatment group	Metabolic activity (%)	Cell number (%)
Vehicle	100.0 ± 2.0	100.0 ± 9.4
IL-1β	102.6 ± 1.1	107.6 ± 19.8
3 μM LY294002	99.9 ± 6.1	69.7 ± 4.6
3 μM LY294002 + IL-1β	99.2 ± 4.2	92.1 ± 9.4
10 μM LY294002	85.8 ± 5.8	83.1 ± 8.1
10 μM LY294002 + IL-1β	87.1 ± 5.1	65.4 ± 8.1
0.3 μM Stattic	124.5 ± 6.5 **	125.6 ± 17.1
0.3 μM Stattic + IL-1β	130.6 ± 6.9 ###	97.7 ± 9.3
3 μM PP1	102.9 ± 3.9	105.7 ± 12.7
3 μM PP1 + IL-1β	96.8 ± 1.6	89.8 ± 11.3
10 μM PP1	85.1 ± 4.2	60.1 ± 10.8
10 μM PP1 + IL-1β	64.9 ± 5.9 ###	45.9 ± 6.9 ##
Vehicle	100.0 ± 1.9	100.0 ± 6.8
LPS	90.8 ± 4.9	99.3 ± 19.5
3 μM LY294002 + LPS	94.9 ± 5.8	89.4 ± 12.0
10 μM LY294002 + LPS	82.1 ± 3.8	71.9 ± 10.1
0.3 μM Stattic + LPS	132.7 ± 8.3 ###	102.5 ± 9.5
3 μM PP1 + LPS	93.9 ± 2.6	80.4 ± 6.7
10 μM PP1 + LPS	65.9 ± 5.5 ##	52.0 ± 9.4 #

Supplementary Table S3: Effect of siRNA treatment on metabolic activity and cell number of HCAEC under IL-1β- or LPS-stimulated conditions. Opti-MEM™ I Reduced Serum Medium, Lipofectamine™ RNAiMAX and the desired siRNA were mixed in a solution and incubated for 20 min at room temperature to form a complex. HCAEC were transfected with a final siRNA concentration of 10 nM each. The cells were allowed to adhere for 24 h. Stimulation of 10 ng/mL IL-1β or 1 μg/mL LPS or vehicle was carried out for 24 h. Subsequently, metabolic activity was determined by WST-1 assay and cell number by crystal violet staining. Cells treated with vehicle were used as control (set as 100%). Data are presented as means ± SEM of $n = 9$ (3 independent experiments). ## $p \leq 0.01$, ### $p \leq 0.001$ vs. corresponding stimulated control; one-way ANOVA plus Bonferroni post hoc test.

Treatment group	Metabolic activity (%)	Cell number (%)
Vehicle + non siRNA	100.0 ± 3.3	100.0 ± 7.3
IL-1β + non siRNA	112.2 ± 5.2	105.6 ± 10.0
IL-1β + VCAM-1 siRNA	92.1 ± 3.2 ##	94.8 ± 6.2
IL-1β + ICAM-1 siRNA	105.5 ± 5.6	96.5 ± 9.4
Vehicle + non siRNA	100.0 ± 2.3	100.0 ± 3.4
LPS + non siRNA	106.8 ± 2.1	164.8 ± 27.8
LPS + VCAM-1 siRNA	86.7 ± 3.7 ###	143.2 ± 16.1
LPS + ICAM-1 siRNA	100.0 ± 4.8	156.2 ± 24.4
